# Gut-Lung Microbiota Axis Shapes the Immune Microenvironment and Immunotherapeutic Response in Lung Cancer

**DOI:** 10.7150/ijbs.126977

**Published:** 2026-02-01

**Authors:** Yao Liu, Sidao Wang, Xuan Xiang, Yiheng Du, Qianqian Xue, Yiran Niu, Wenbei Peng, Linlin Ye, Qiong Zhou

**Affiliations:** 1 Department of Respiratory and Critical Care Medicine, The Central Hospital of Wuhan, Tongji Medical College, Huazhong University of Science and Technology, Wuhan, China.; 2 Department of Respiratory and Critical Care Medicine, Union Hospital, Tongji Medical College, Huazhong University of Science and Technology, Wuhan, China, 430000.

**Keywords:** lung cancer, gut-lung axis, immunotherapy, microbiota

## Abstract

The gut-lung axis microbiota plays a pivotal role in shaping the tumor immune microenvironment (TIME) and regulating immunotherapeutic responses in lung cancer. This review highlights that pulmonary and gut microbial dysbiosis drives lung cancer development through inducing chronic inflammation, remodeling the immune microenvironment, and reprogramming metabolism. Lung cancer patients exhibit distinct microbial signatures characterized by altered microbiotal diversity and enrichment of specific taxa like Streptococcus, Veillonella, and Bacteroidetes in the airways, along with gut microbial shifts involving decreased Firmicutes/Bacteroidetes ratio. These microbial alterations promote tumor progression via activation of pro-inflammatory pathways (e.g., interleukin-17 (IL-17)/interleukin-23 (IL-23) axis) and suppression of antitumor immunity.Notably, the gut-lung microbiome exerts a profound impact on immunotherapeutic efficacy: responders are enriched with beneficial microbes like Akkermansia muciniphila and Bifidobacterium that enhance CD8⁺ T cell responses, while non-responders show elevated levels of Gammaproteobacteria and Fusobacterium associated with immunosuppression. Regulatory mechanisms include systemic immune modulation by microbial metabolites such as short-chain fatty acids, as well as activation of key signaling pathways including cGAS-STING and CD40L-CD40/NF-κB. Emerging translational applications encompass lung cancer diagnosis and immunotherapeutic response prediction via microbial biomarkers, as well as therapeutic interventions including fecal microbiota transplantation (FMT) and probiotic supplementation. Future studies should clarify microbe-host interaction mechanisms and develop personalized microbiota-based strategies to overcome immunotherapy resistance, offering the potential to revolutionize precision oncology through integrating microbiota modulation with conventional therapies.

## Introduction

**Figure 1:**
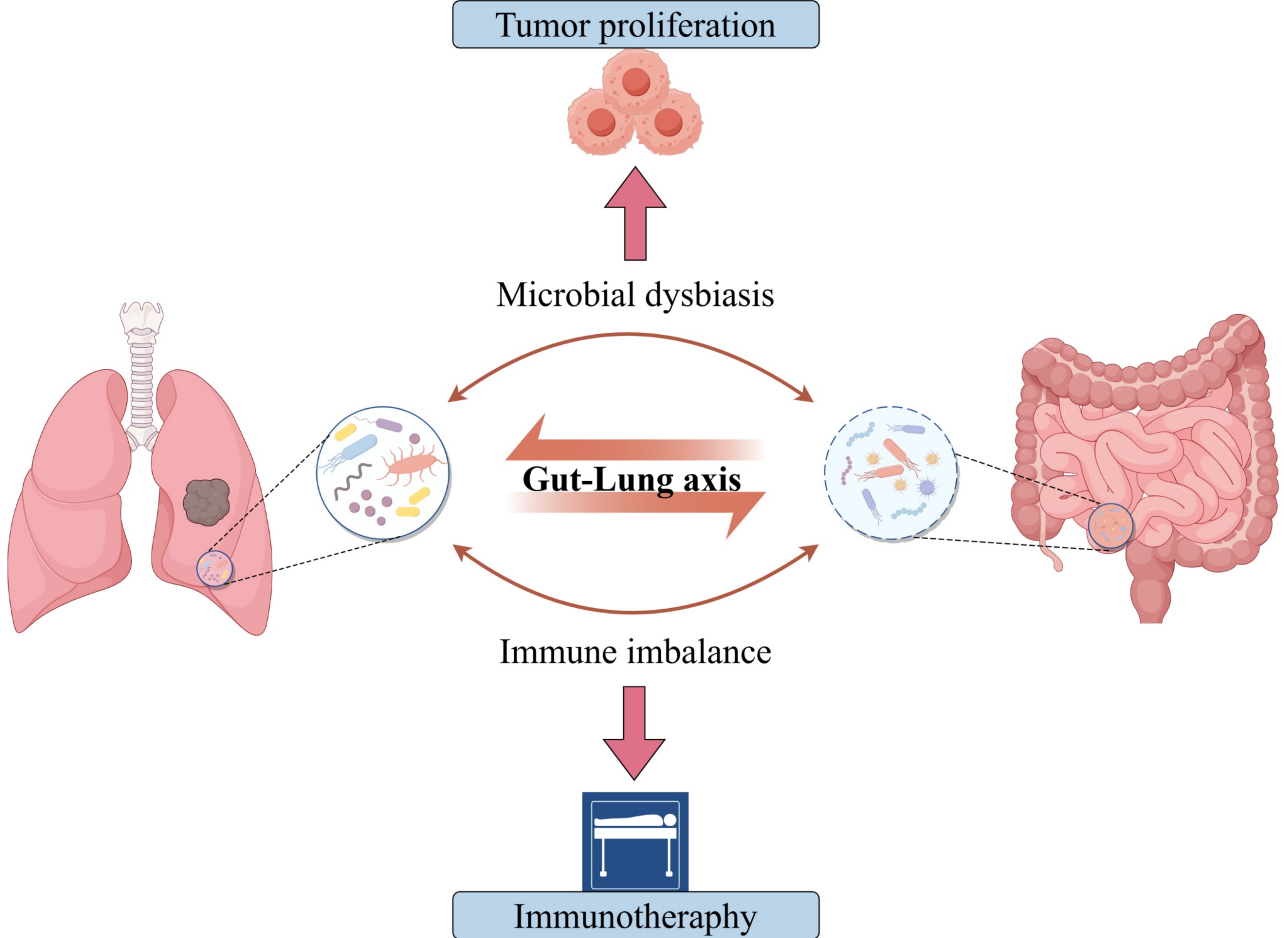
** The Gut-Lung Microbiota Axis Dysbiosis Regulates Lung Cancer Progression and Immunotherapeutic Response.** Emerging evidence positions gut-lung microbiota axis perturbation as an overarching regulator spanning from lung cancer initiation to immunotherapy outcomes, wherein compartmentalized dysbiosis rewires host-tumor microenvironments via multi-organ crosstalk.

Emerging epidemiological data reveal that in 2023, lung cancer accounted for approximately 2.30 million new cases and 2.04 million deaths globally, ranking first among all malignant neoplasms 1. Projections indicate that in 2025, the United States will record 226,650 incident cases of lung cancer, representing approximately 11.1% of all new cancer diagnoses, with 124,730 attributable deaths constituting about 20.2% of total cancer mortality 2. This highlights an urgent need for revolutionary breakthroughs in current diagnostic and therapeutic approaches. Immune checkpoint inhibitors (ICIs) have revolutionized the therapeutic landscape for lung cancer. Compared to conventional chemotherapy and targeted therapies, ICIs restore T-cell function by blocking immune checkpoint proteins including programmed death 1 (PD-1), programmed death-ligand 1 (PD-L1), and cytotoxic T-lymphocyte-associated protein 4 (CTLA-4), thereby significantly prolonging survival in subsets of advanced non-small cell lung cancer (NSCLC) patients 3. Nevertheless, immunotherapy remains hindered by significant constraints, with less than 50% of patients attaining sustained clinical responses from ICIs monotherapy while the majority confront primary or acquired resistance 4, 5. Furthermore, the accurate quantification of PD-L1 expression persists as a challenging task owing to the difficulties in sample acquisition and intratumoral heterogeneity 6, 7. Meanwhile, emerging biomarkers such as tumor mutational burden (TMB) are unable to completely surmount the challenge of precise patient stratification 8. The microbiota-cancer interplay has emerged as a promising therapeutic avenue (Figure 1). Landmark studies first demonstrated colonic commensals promote colorectal tumorigenesis 9, subsequently establishing microbial regulation of chemotherapy/immunotherapy responses 10, 11 and CTLA-4 blockade efficacy 12. Microbiota now constitute key immunotherapy modulators, with their roles in lung cancer progressively elucidated. Research findings suggest that the lungs are not in a sterile state, and airway microbiome dysbiosis can increase the risk of chronic respiratory diseases 13, 14. Particularly in lung cancer, dysbiotic microbiota regulates tumorigenesis through host interactions and remodels the tumor immune microenvironment (TIME) via mechanisms such as pro-inflammatory factor release, induction of immune tolerance, and disruption of barrier function 15. Notably, this comprehension has extended from the pulmonary realm to the domain of the gut-lung axis, indicating that the distal gut microbiota also assumes a crucial regulatory function in pulmonary diseases 16, 17. The lungs and the gut share a common embryogenic origin, exhibit similarities in structure and function, and possess comparable mucosal immune characteristics^.^ The bidirectional regulatory mechanism between the lungs and the gut is mainly accomplished through the bidirectional migration of immune cells, mediators, and microbiota 18, 19. Furthermore, the microbiome has the potential to function as a novel biomarker for prognosticating the efficacy of ICIs and as a target for enhancement. Interventions such as fecal microbiota transplantation and probiotic supplementation can significantly enhance antitumor immunotherapy responsiveness 20, 21. These findings provide new strategies for overcoming resistance to tumor immunotherapy.

Building on these insights, this study systematically investigates how lung-gut microbiota dysbiosis affects lung carcinogenesis and immunotherapy response. By characterizing microbiota lineage changes and their immune-metabolic networks in tumor microenvironment (TME) regulation, this study proposes novel strategies for microbiota-assisted precision diagnostics and immunotherapy enhancement.

## Clinical Evidence Implicating Gut-Lung Microbiota Dysbiosis in Lung Cancer Pathogenesis

### Characterization of Microbial Diversity Alterations in Lung Cancer

In health, the dynamic equilibrium of the lung microbiome relies predominantly on the immigration-elimination balance, exhibiting a progressive decline in community richness as distance from the upper respiratory tract source community increases. Impaired airway clearance, increased microbial colonization, and enhanced local proliferation collectively alter lung microbiota diversity in lung cancer patients 13. Several studies based on 16S rRNA sequencing, next-generation sequencing (NGS), whole metagenomic sequencing (WMS), etc., have indicated a decrease in α-diversity among lung cancer patients (Table 1, Table S1). These findings show reduced microbial richness across various sample types, including tumor tissues, airway samples such as bronchoalveolar lavage fluid (BALF), bronchial brushings, and sputum, when compared to adjacent non-tumor tissue, non-cancer controls, or healthy populations 22-27. Notably, tumor tissues from patients with recurrence exhibited significantly lower α-diversity compared to non-recurrent cases, and this metric may serve as an independent predictor for recurrence-free survival following pulmonary resection 28. In addition, advanced-stage (III-IV) patients exhibited decreased α-diversity in sputum samples compared to early-stage (I-II) cases 29, while oral swab samples with higher α-diversity were associated with reduced cancer risk 30. Conversely, some studies have reported increased α-diversity in tumor tissues 31-33, BALF 34, 35, and even saliva samples 36 from lung cancer patients compared with control groups, which predominantly comprised patients with specific respiratory diseases such as emphysema-only cases, tuberculosis patients, or pneumonia patients. It is worth noting that several studies have not detected any significant differences in microbial α-diversity between the two groups 37-40. Unlike findings in the lungs, most studies indicate no significant difference in gut microbial α-diversity between lung cancer patients and non-cancer controls, while some reports observe decreased microbial α-diversity 41, alongside increased fungal diversity in patients 42 (Table 2, Table S2). Furthermore, impaired pulmonary ventilation was found to be positively associated with the α-diversity of gut microbiota in patients with lung cancer, indicating that a decline in pulmonary function correlates with an increased microbial diversity 43. The microbial composition significantly differs between lung cancer patients and healthy controls. Significant β-diversity distinctions have been consistently observed between groups across multiple sampling sites, particularly in lung (BALF, tumor tissue) and gut (stool samples) 23, 26, 27, 31, 32, 36, 37, 42, 44-54, with variations further associated with tumor histology, TNM stages 29, 38, and recurrence outcomes 28. However, some studies failed to replicate these findings in BALF or stool samples 25, 30, 34, 35, 39, 40, 55-57. Evidence suggests significant associations between microbial diversity (e.g., α-/ β-diversity) and lung cancer, although inconsistencies remain across studies. Overall, compared to non-cancer controls, lung cancer patients exhibit characteristic alterations in the gut-lung microbiota, typically showing decreased α-diversity and significant β-diversity variations. The concept of "global restructuring of microbial community composition and function" may provide a unifying framework to explain the heterogeneity observed across different studies. Future research should employ larger sample sizes and more comprehensive analytical approaches to systematically characterize the structural and functional features of pulmonary and intestinal microbiota in lung cancer patients.

### Lung Cancer-Associated Alterations in Microbial Compositional Profiles

Multiple studies have demonstrated significant differences in lung microbiota composition between lung cancer patients and non-cancer controls (Table S3). Analyses of oral (saliva), lower respiratory tract (BALF/bronchial washing fluid/bronchial brushes), and tumor tissue samples consistently revealed enrichment of *Firmicutes* in LC patients, accompanied by elevated *Bacteroidota* and *Proteobacteria* in tumor tissues 32, 33, 39, 45, 55. Notably, *Actinobacteria* showed marked enrichment in airway samples from synchronous multiple primary lung cancer (sMPLC) patients 37.

Additionally, the lower respiratory tract (LRT) microbiota of lung cancer patients exhibits distinct genus-level alterations. Studies demonstrate significant enrichment of typical supraglottic taxa including *Streptococcus*, *Veillonella*, Prevotella, and *Rothia* in the lower airways, concomitant with elevated rRNA gene concentrations in BALF 27, 44, 56. These microbial shifts correlate strongly with enhanced pulmonary inflammation, as evidenced by increased neutrophil and lymphocyte counts and exhaled nitric oxide (eNO) levels in BALF 58. The microbial composition exhibits significant modulation by distinct clinical characteristics such as tumor node metastasis (TNM) stage, distant metastasis, pathological subtype, and epidermal growth factor receptor (EGFR) mutation status. Notably, *Thermus* predominates in advanced-stage (IIIB/IV) tumors, *Legionella* shows strong associations with metastatic progression, and *Acidovorax* enrichment characterizes smoking-related squamous cell carcinomas, particularly in cases with tumor protein p53 (TP53) mutations 31.

Significantly, *Parvimonas* shows specific enrichment in EGFR-mutant lung adenocarcinoma (LUAD) 29. Fungal taxa including *Aspergillus*, *Agaricomycetes*, and *Blastomyces* are also overrepresented in tumor tissues, though their mechanistic roles in carcinogenesis remain to be elucidated 59, 60. Analysis of stool samples from lung cancer patients revealed a distinct gut microbiome signature (Table S4), characterized by significant enrichment of *Bacteroidetes*, *Proteobacteria*, *Halanaerobiaeota*, and *Desulfobacterota* concurrent with depletion of *Actinobacteria*. At the species level, *Streptococcus anginosus* was significantly enriched in rectal swabs of LUAD patients, whereas *Corynebacterium aurimucosum* predominated in lung squamous cell carcinoma (LUSC) cases 33, 49, 51, 52, 57, 61. Notably, the ratio of *Firmicutes*/*Bacteroidetes* was significantly reduced in the gut microbiota of lung cancer patients 41. Given that *Firmicutes* and *Actinobacteria* facilitate colonic short-chain fatty acids (SCFAs) production, this decline correlates with diminished circulating SCFAs levels 62, 63. Fungal community analysis demonstrated significant enrichment of *Basidiomycota*, *Mortierellomycota*, and *Chytridiomycota* but depletion of Ascomycota in LUAD patients 42. Specific bacterial genera including *Bacteroides*, *Veillonella*, *Megasphaera*, and *Enterococcus*, and fungal genera including *Saccharomyces* and *Aspergillus*, showed significantly increased abundance in stools of lung cancer patients compared to controls. Additionally, building upon previous research findings, this study identified the anaerobic bacteria *Prevotella* and *Veillonella* as shared lung cancer-associated genus across both lung (including BALF, bronchial brushing, and lung tumor tissue samples) and gut (stool samples) sites (Figure S1). These anaerobic bacteria, which demonstrate high abundance and ubiquitous colonization in humans 64, 65, may mediate trans-organ carcinogenesis through the gut-lung axis. Overall, the systematic analysis of microbial dysbiosis in the lungs and gut reveals lung cancer-specific microbial signatures. Alterations in microbiota composition, both in diversity and taxon abundance, demonstrate site-specific patterns with significant clinical associations. This microbe-host interaction contributes to the initiation and progression of lung cancer.

## Potential Mechanisms of Gut-Lung Microbiota in Lung Cancer Pathogenesis

### Lung Microbiota Modulates Local Immune Remodeling and Oncogenic Signaling

**Figure 2:**
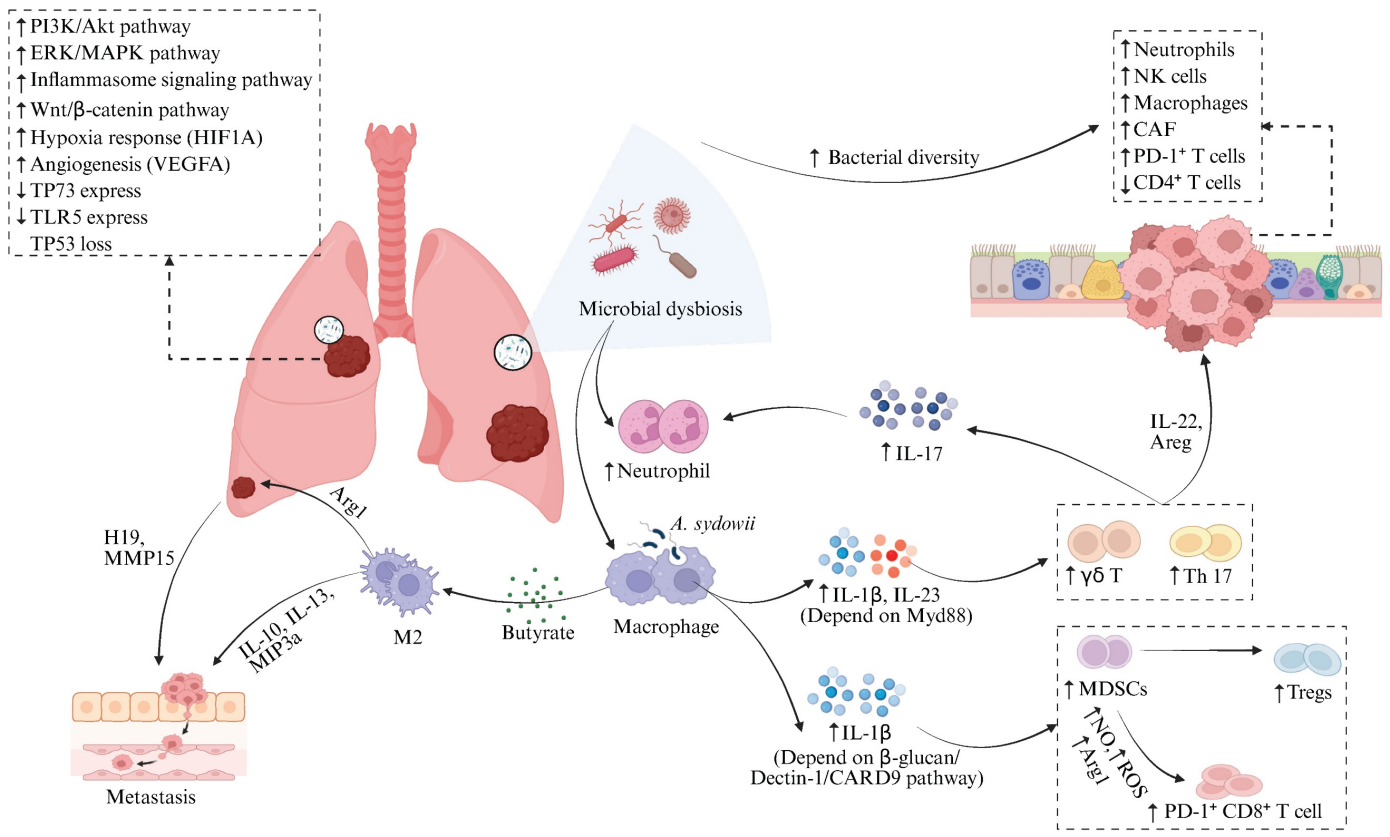
** Pulmonary microbiota dysbiosis drives lung cancer progression through dual mechanisms.** Airway microbiota dysbiosis drives lung cancer progression through multiple mechanisms, including direct activation of ERK/MAPK, PI3K/AKT, and inflammasome signaling pathways in respiratory epithelial cells to promote tumorigenesis; modulation of key gene expression indicated by positive correlations between tumor bacterial burden and CTNNB1 (Wnt/β-catenin), HIF1A (hypoxia response), and VEGFA (angiogenesis), alongside negative correlations with TP73 and TLR5; and immune microenvironment remodeling evidenced by positive associations between bacterial diversity and CAF infiltration or immune exclusion traits, resulting in enrichment of neutrophils, macrophages, and NK cells in high-diversity tumors and closely linked to increased PD-1⁺ T cells and CD4⁺ T cell exhaustion. Specifically, airway dysbiosis induces Myd88-dependent IL-1β/IL-23 release from alveolar macrophages and neutrophils, activating tissue-resident Vγ6⁺Vδ1⁺ γδ T cells to secrete IL-17. This synergizes with Th17 cell responses to drive neutrophilic recruitment and perpetuate pulmonary inflammation. Lung cancer tissue-enriched *Aspergillus sydowii* induces IL-1β via the β-glucan/Dectin-1/CARD9 axis, promoting MDSC and Treg expansion alongside PD-1⁺CD8⁺ T cell accumulation, fostering immunosuppression. *Roseburia*-derived butyrate upregulates non-coding RNA H19, increasing MMPs like MMP15 to facilitate metastasis; concurrently, butyrate polarizes M2 macrophages to secrete IL-10, IL-13, and MIP3α, enhancing tumor migration and invasion. Figure 2 was created using BioRender and holds the appropriate license. Amphiregulin, Areg; Arginase-1, Arg1; Cancer-associated Fibroblast, CAF; Caspase recruitment domain-containing protein 9, CARD9; Cytotoxic T Lymphocyte, CTL; Extracellular Signal-Regulated Kinase, ERK; Interleukin, IL; Matrix Metalloproteinases, MMPs; Macrophage Inflammatory Protein-3 alpha, MIP-3α; Myeloid-Derived Suppressor Cells, MDSCs; Mitogen-Activated Protein Kinase, MAPK; Natural Killer cells, NK; Nitric Oxide, NO; Programmed Cell Death Protein 1, PD-1; Phosphoinositide 3-Kinase, PI3K; Reactive Oxygen Species, ROS; Regulatory T Cells, Tregs; Toll-Like Receptor, TLR.

The lung microbiota contributes to the pathogenesis of lung cancer through various mechanisms, including inducing local inflammatory responses, remodeling the immune microenvironment, disrupting cellular metabolism, and modulating gene expression 66-69 (Figure 2). Research finding has demonstrated a marked dysbiosis in the lower respiratory tract of lung cancer patients, which is prominently characterized by the abnormal colonization of supraglottic microbiota, such as *Streptococcus* and *Veillonella* 44. Notably, *Veillonella parvula* shows specific enrichment in the lower airways and exhibits a significant correlation with poor prognosis in advanced-stage (TNM IIIB-IV) LUAD patients, as evidenced by notably decreased survival and increased tumor burden 38. Additionally, advanced pulmonary adenocarcinoma correlates with an airway microbiota featuring a "high-burden, low-diversity" characteristic 70. In this context, the elevated bacterial load positively correlates with tumor burden, further corroborating the significant association between microbial dysbiosis and lung cancer progression. Airway microbiota dysbiosis promotes lung cancer progression through multifaceted mechanisms. The dysbiotic microbiota activates the extracellular signal-regulated kinase/mitogen-activated protein kinase (ERK/MAPK) and phosphoinositide 3-kinase/protein kinase B (PI3K/AKT) signaling pathways in respiratory epithelial cells, enhancing cellular proliferation, survival, differentiation, and invasive capacity to drive tumorigenesis 71. Microbiota-derived SCFAs may function as crucial mediators, driving oncogenesis through insulin-like growth factor 1 (IGF-1) signaling and M2-like macrophage polarization. These processes synergistically upregulate the PI3K and MAPK signaling pathways 72. Microbiota dysbiosis remodels the TIME by activating interleukin-17A (IL-17A), interleukin-1 (IL-1), interleukin-6 (IL-6), and inflammasome signaling pathways, which results in the expansion of T-helper (Th17) cells, the upregulation of PD-1⁺ T cells, and the exhaustion of CD4⁺ T cells within the tumor tissue 38, 73, 74. Notably, airway microbiota imbalance stimulates alveolar macrophages and neutrophils to produce interleukin-1β (IL-1β) and IL-23 via Myd88-dependent myeloid signaling within tumor tissues, thereby inducing the proliferation and activation of tissue-resident Vγ6+Vδ1+ γδ T cells 70. Activated γδ T cells serve as the principal source of IL-17 in tumor-bearing lungs. In combination with Th17 cells, they are the main producers of IL-17 75. The upregulated IL-17 further promotes neutrophil infiltration and chronic pulmonary inflammation 76. Accumulating evidence suggests the functional importance of neutrophils in tumor immunity, progression, and response to immunotherapy 77.

Collectively, the enhanced pulmonary inflammation and diminished antitumor immune surveillance contribute to the establishment of a local pro-tumorigenic immune environment 78. Similarly, the intratumoral microbiome within lung cancer tissue engages in disease regulation. Bacterial burden augmented from tumor-adjacent normal lung tissue and tertiary lymphoid structures to tumor tissue and finally to the airways, implying airway-mediated invasion of intratumoral bacteria. Spatial transcriptomic analysis 79 revealed that bacteria within lung tumors were predominantly enriched in tumor cells, with their burden showing a strong positive correlation with the expression of genes involved in the Wnt/β-catenin signaling pathway (catenin beta 1, CTNNB1), hypoxia response (hypoxia-inducible factor 1 alpha, HIF1A), and angiogenesis (vascular endothelial growth factor A, VEGFA). These dysregulated gene expressions significantly contribute to tumor initiation and progression 80-83. Moreover, the bacterial burden on these tumor cells showed a negative correlation with genes associated with cell cycle arrest and apoptosis (tumor protein 73, TP73) 84 and pattern recognition (Toll-like receptor 5, TLR5) 85. These findings suggest that tumor-associated bacteria promote tumor growth. Nevertheless, these bacteria fail to augment the immunogenicity of tumor cells, as there exists no notable correlation between bacterial burden and the expression of genes associated with the antigen presentation pathway 79. The fungus *Aspergillus sydowii* is significantly enriched in LUAD tumor tissues, which promotes cancer progression by inducing an immunosuppressive TME. This phenomenon is manifested by the expansion and activation of myeloid-derived suppressor cells (MDSCs) 86 and regulatory T cells (Tregs) 87^,^ along with the upregulation of immunosuppressive gene signatures related to myeloid leukocyte chemotaxis, reactive oxygen species (ROS) metabolic and IL-1β production 88-90.* Aspergillus sydowii* activates MDSCs through the secretion of IL-1β via the β-glucan/Dectin-1/caspase recruitment domain-containing protein 9 (CARD9) axis, thereby inhibiting the activity of cytotoxic T lymphocytes and promoting the accumulation of PD-1^+^CD8^+^ T cells 24. This mechanism contributes to patient immunosuppression and unfavorable prognosis. Fungal enrichment (e.g., *Alternaria alternata*, *Malassezia globosa*) in pancreatic ductal adenocarcinoma (PDAC) drives Kras^G12D^-dependent activation of the dectin-1-mediated Src-Syk-CARD9 pathway, resulting in interleukin-33 (IL-33) secretion. This recruits and polarizes Th2 cells and innate lymphoid cells (ILC2s), ultimately accelerating tumor progression via elevated interleukin-4 (IL-4), interleukin-5 (IL-5), and interleukin-13 (IL-13) production. Antifungal therapy markedly attenuates PDAC progression 91, 92. *Alternaria*-derived serine protease activity induces rapid IL-33 release from lung epithelium, driving robust Th2 inflammation that contributes to early pulmonary inflammation 93; however, its role in lung cancer remains unexplored despite reports of *Alternaria* enrichment in tumor tissues of NSCLC patients 46.

Emerging evidence indicates that microbiota may contribute to lung cancer recurrence and metastasis. The butyrate-excreting bacterium* Roseburia* could promote tumor recurrence and metastasis 28, as low butyrate levels inhibit histone deacetylase 2 (HDAC2), thereby enhancing histone H3 Lysine 27 (H3K27) acetylation and upregulating the non-coding ribonucleic acid (RNA) H19. Subsequently, H19 upregulates multiple matrix metalloproteinases (MMPs) 94, 95, including MMP15 which plays key roles in NSCLC metastasis 96. The Cancer Genome Atlas (TCGA) database analysis reveals MMP15 overexpression is associated with advanced TNM stages and shorter recurrence-free survival (RFS) in NSCLC patients. Beyond the H19-MMP pathway, low-dose butyrate induces M2 macrophage polarization, enhancing secretion of interleukin-10 (IL-10), IL-13, and MIP-3α and thereby promoting tumor cell migration and invasion 28, 97, 98. Microbes remodel the immune microenvironment and signaling in metastatic tumors. Specifically, higher bacterial diversity is associated with elevated signatures of cancer-associated fibroblast (CAF) infiltration and immune exclusion. Furthermore, lung metastatic with rich microbial diversity exhibit increased infiltration of innate immune cells such as neutrophils, natural killer (NK) cells, macrophages, and Tregs, along with an enhanced signaling through EGFR. Specifically, there is a positive association between *Bifidobacterium* abundance and NK cells (S2 state) 99. This result aligns with previous study 100, demonstrating that *Bifidobacterium* restores NK cell frequency and enhances tumor immunity within the TME. Collectively, these findings demonstrate that microorganisms within lung cancer cells contribute to TME modulation, promoting tumor progression and immune evasion, underscoring their tumor-promoting role.

### Gut Dysbiosis Drives Systemic Immune Modulation and Metabolic Reprogramming

**Figure 3:**
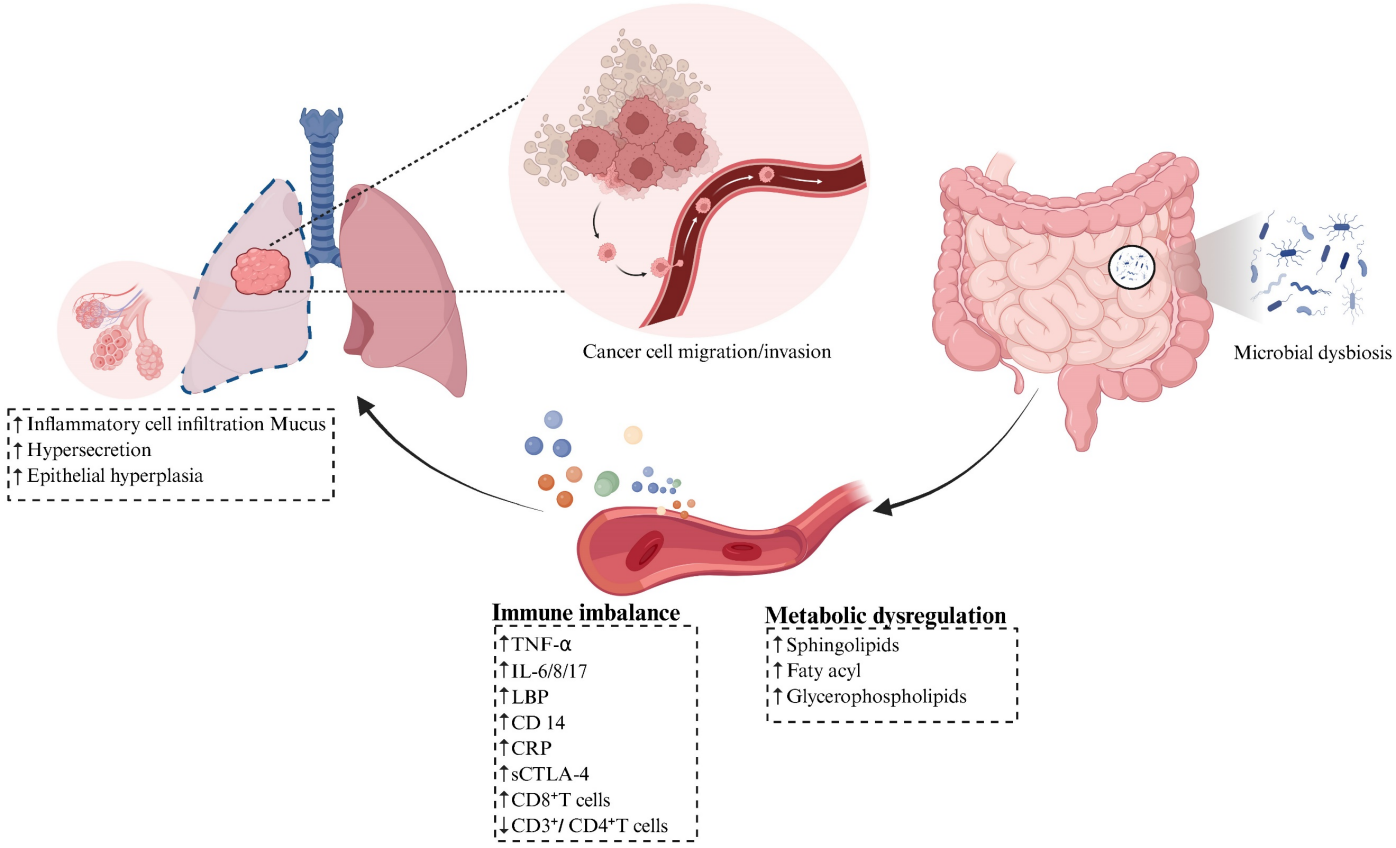
** Gut Microbiota Modulates the Progression of Lung Cancer.** Gut microbial dysbiosis promotes lung cancer progression by remodeling systemic immunity and metabolism. Fecal microbiota transplantation (FMT) from lung cancer patients induces systemic immune imbalance, specifically manifested by significant elevation of serum pro-inflammatory mediators (TNF-α, IL-8), endotoxin-related markers (LBP, CD14), and CRP, alongside increased peripheral blood CD8⁺ T cells and decreased CD3⁺/CD4⁺ T cells, accompanied by inflammatory cell infiltration in alveolar cavities, hyperactive mucus secretion in lung tissue, and abnormal epithelial hyperplasia. Compared to healthy controls, lung cancer patients exhibit synchronized elevation of plasma pro-inflammatory/immunosuppressive factors such as IL-6, IL-17, and sCTLA-4. Gut microbial dysbiosis in lung cancer patients further causes abnormal lipid metabolism, characterized by significantly elevated peripheral levels of sphingolipids, fatty acyl and glycerophospholipids, indicating hyperactive lipid metabolism and oxidative stress status. Figure 3 was created using BioRender and holds the appropriate license. Binding protein, LBP; CD14 molecule, CD14; C-reaction protein, CRP; Interferon-gamma, IFN-γ; Tumor necrosis factor-α, TNF-α; Soluble cytotoxic T-lymphocyte-associated protein 4, sCTLA-4.

Gut dysbiosis plays a crucial role in driving the pathogenesis of lung cancer through the remodeling of systemic immunity and the reprogramming of host metabolic homeostasis, consequently accelerating tumor proliferation, invasion, and metastatic progression (Figure 3). Experimental evidence shows fecal microbiota transplantation from lung cancer patients into tumor-bearing Lewis lung carcinoma C57BL/6 mice, or monocolonization with lung cancer-enriched Prevotella copri, induces systemic immunodysregulation. This manifests through elevated serum levels of proinflammatory mediators such as tumor necrosis factor-α (TNF-α) 101 and interleukin-8 (IL-8) 102, along with endotoxin biomarkers including lipopolysaccharide binding protein (LBP) and CD14 molecule (CD14), as well as increased C-reaction protein (CRP). Concurrently, peripheral CD3⁺/CD4⁺ T cells decline, while CD8⁺ T cells rise. These inflammatory factors induced alveolar inflammatory cell infiltration, excessive mucus secretion in lung tissues, and abnormal epithelial hyperplasia. Critically, nervonic acid/all-trans retinoic acid intervention reverses these pathologies, demonstrating reversible microbiota-host pathogenic interplay 57. Compared with healthy controls, lung cancer patients exhibit synchronous elevation of pro-inflammatory and immunosuppressive mediators, particularly IL-6, IL-17, and soluble CTLA-4 (sCTLA-4) in peripheral blood. This triad establishes a self-perpetuating 'microbiota-metabolite-immune' pathological circuit 48. Ni et al. 53 identified significant enrichment of *Agathobacter*, *Blautia*, and *Clostridium* genera along with *Muribaculaceae* family in the gut microbiota of early-stage NSCLC patients. This dysbiosis coincided with abnormally elevated peripheral levels of sphingolipids, fatty acyl, and glycerophospholipids. Further analyses revealed positive correlations between *Clostridium* abundance and multiple glycerophospholipids, while *Muribaculaceae* abundance was associated with specific phospholipid metabolism perturbations and fatty acyl. Moreover, an independent study demonstrated that aberrantly enriched *Clostridioides* and *Synergistes* genera exhibit significant positive correlations with peripheral glycerophospholipid metabolism in lung cancer cohorts, suggesting these microbes may drive pathogenesis through lipid metabolic reprogramming 52.

Patients with lung cancer consistently exhibit dysregulated lipid metabolism, manifested by heightened levels of acylcarnitines and phospholipids, alongside enhanced lipid metabolic activity and oxidative stress. Notably, glycerophospholipids and sphingolipids serve as essential membrane components, facilitating transmembrane signaling and transport. Phospholipids additionally function as signaling molecules, allosteric enzyme activators, and central regulators of energy metabolism, and their dysregulation directly correlates with tumor progression 103. Moreover, sphingolipids mediate inflammatory signals and the release of pro-inflammatory cytokines, thereby modulating intracellular complement activation. This cascade induces NOD-like receptor family, pyrin domain-containing 3 (NLRP3) inflammasome assembly, triggering caspase-1-dependent IL-1β maturation that stimulates malignant cell dissemination 104. Impaired functional activity of intestinal microbiota constitutes the pivotal mechanism underlying microecological dysbiosis in lung cancer. Metagenomic analyses reveal significantly diminished expression of proteins involved in amino acid transport/metabolism, coenzyme transport/metabolism, cell cycle control, and cell division within the gut microbiome of lung cancer patients 49. Furthermore, these patients exhibit markedly reduced abundance in energy metabolism pathways and ATP-binding cassette (ABC) transporter systems compared to healthy controls 41. This pervasive functional suppression compromises bacterial proliferation and colonization capacity, destabilizes microbial communities, and perpetuates metabolic derangement with concomitant immune dysregulation. Targeting the aforementioned mechanisms 105, modulating microbiota-host interactions demonstrates therapeutic potential. In Lewis lung cancer (LLC) tumor-bearing models, oral administration of polysaccharopeptides (PSP) significantly suppressed pro-tumorigenic mediators, specifically leukotriene B₄ (LTB₄), leukotriene D₄ (LTD₄), and leukotriene E₄ (LTE₄), through inhibition of FcεRI signaling and downstream arachidonic acid metabolism. These leukotrienes drive tumor progression through increased vascular permeability, inflammatory cascade activation, TNF-α/IL-1/IL-6/IL-2 overexpression, and amplified Th2-polarized pulmonary inflammation 106-108. Crucially, PSP concurrently restores microbial equilibrium while inhibiting this pathway, thereby suppressing lung cancer proliferation.

### Integrated Mechanisms of the Gut-Lung Axis Mediated by Metabolic Signaling and Immune Trafficking

A well-established gut-lung axis mediates bidirectional crosstalk through microbial communities and their metabolites, profoundly influencing lung cancer pathogenesis and progression. Mao et al. 109 found that the development of lung cancer is inversely correlated with the microbial diversity in the lung and gut compartments. This dysbiosis coincides with lung tissue accumulation of pro-inflammatory mediators (leukotriene B4 and prostaglandin E2) and concomitant elevation of intestinal anti-inflammatory metabolites (resolvins and isohumulones). Important evidence indicates that gut-lung axis microbial translocation plays a pivotal role in lung disease pathogenesis 110-112. Specifically, gut microbiota dysbiosis facilitates systemic translocation of gut microbiota to lung tissues via circulatory pathways, which promotes lung cancer progression by disrupting lung microbial homeostasis and potentiating inflammatory signaling cascades 113-115. The gut communicates with distant organs including the lungs through microbiota and their derived metabolites, establishing intimate cross-talk. Short-chain fatty acids (SCFAs), including representative molecules such as butyrate, acetate, propionate and valerate, are produced through the fermentation of dietary fibre by the intestinal microbiota. Butyrate can promote the extrathymic generation of regulatory T cells (Treg) by activating G protein-coupled receptor signaling on the cell membrane or by inhibiting histone deacetylase activity 116. Consequently, it suppresses inflammation, modulates epithelial barrier function, and regulates mucosal and systemic immunity 117. Acetate and propionate enhance the integrity of the intestinal barrier, reduce bacterial translocation, and alleviate systemic inflammatory responses 118^.^ Previous study has demonstrated that *Bacteroides* in the gut can enhance the anti-tumour activity of CD8^+^ T cells by producing the metabolite acetate, thereby inhibiting the progression of breast cancer 119. Building on this, Luu et al. further identified that valerate can act as a novel metabolic regulatory molecule for the treatment of autoimmune diseases by reprogramming lymphocyte metabolic activity to promote the secretion of the anti-inflammatory cytokine IL-10 and suppress the differentiation of pro-inflammatory Th17 cells 120^.^ Similarly, gut-derived SCFAs can exert distal regulatory effects on lung diseases. Robust epidemiological evidence 121 indicates that antibiotic use in early life increases the risk of developing asthma in adulthood, a phenomenon that may be related to antibiotic-induced disruption of the gut microbiota and consequent reduction in butyrate production. Butyrate can suppress allergic Th2-type immune responses by downregulating the migratory and activation capacity of dendritic cells (DCs) 122. Clinical studies have also shown that individuals with higher levels of butyrate-producing bacteria in the gut have a significantly reduced risk of lower respiratory tract viral infections 123. This protective effect is closely associated with the ability of propionate and butyrate to effectively enhance the antiviral immune function of pulmonary CD8^+^ T cells. Beyond SCFAs, the gut microbiota also produce a variety of other metabolites, such as bile acids and amino acid derivatives, which may also influence pulmonary immunity and inflammation via the gut-lung axis 124. For example, gut-derived indole compounds can be transported to the lung via the bloodstream, where they influence T lymphocyte differentiation and the polarization status of alveolar macrophages, thereby altering immune homeostasis and the level of inflammatory responses in lung tissue 125, 126. Beyond microbial metabolites, immune cells mediate bidirectional gut-lung axis regulation. Virus-activated lung CD4⁺ T cells upregulate CCR9 chemokine receptors and migrate to the intestinal mucosa, where Interferon-gamma (IFN-γ) secretion disrupts microbiota homeostasis 127. Concurrently, gut innate lymphoid cells (ILCs) can extravasate into lung tissues through lymphatic or systemic circulation, where they modulate regional inflammation and coordinate synchronized immune responses between the two organs 128. This form of cross-organ communication not only remotely remodels the lung immune microenvironment and affects lung cancer progression but also offers novel targets for improving responses to lung cancer immunotherapy by modulating the gut microbiota.

## Gut-Lung Microbiota Axis Modulates Antitumor Immunotherapy

### Clinical relevance: Microbial signatures predict the efficacy of immunotherapy response

Multiple studies have demonstrated that specific gut and lung microbial signatures are closely associated with the efficacy of ICIs (Table 3). In ICI responders, the lung microbiota characterized by significantly increased α-diversity and enrichment of commensal taxa such as the *Bacteroidetes* and *Veillonella dispar*, these features are positively correlated with higher objective response rates (ORR), including complete response (CR), partial response (PR), and stable disease (SD) incidence 129, 130. In contrast, the lung microenvironment of non-responders is predominantly occupied by *Gammaproteobacteria* and *Fusobacterium,* whose aberrant overgrowth is strongly associated with shorter progression-free survival (PFS) and overall survival (OS). Among these, the abundance of *Gammaproteobacteria* is negatively correlated with PD-L1 expression, whereas higher *Fusobacterium* abundance is associated with markedly reduced intratumoral cytotoxicity, IFN-γ levels, and expression of major histocompatibility complex (MHC) class II genes 99, 131. The gut microbiota exhibits significant prognostic value. Characteristic microbial signatures including *Akkermansia muciniphila* (*A. muciniphila*), *Clostridium butyricum* MIYAIRI 588, and *Bifidobacterium bifidum* are significantly associated with prolonged PFS and OS 132-136. Notably, the survival benefit conferred by *A. muciniphila* exhibits a distinct nonlinear relationship, with optimal therapeutic benefits observed at intermediate relative abundance (0%-4.8%). This range frequently co-occurs with other beneficial taxa such as *Ruminococcus*, *Faecalibacterium*, and *Bifidobacterium*, which have been established to correlate with favorable ICI outcomes 134, 137-139, underscoring microbiota interactions. Conversely, depletion or excessive proliferation of *A. muciniphila* typically coincides with enrichment of pathogenic taxa including *Veillonella*, *Actinomyces*, and *Clostridium*, which are associated with poorer prognosis 38, 140. Furthermore, the strain *Hominenteromicrobium* YB328 has been found to be significantly enriched in the gut of responders and has been shown to act synergistically with anti-PD-1 therapy to potently inhibit tumor growth 141. Clinical evidence indicates that antibiotic use disrupts host microbial homeostasis and may even lead to the overgrowth and recolonization of pathogenic bacteria (e.g., *Enterocloster*), thereby significantly reducing the efficacy of ICIs 132, 142-144. Importantly, *Clostridium butyricum* MIYAIRI 588 has been shown to reverse antibiotic-induced dysbiosis and consequently restore survival in lung cancer models 133, 145. Furthermore, gut microbial metabolism significantly influences the therapeutic efficacy of ICIs. Metabolomics studies have confirmed that in NSCLC patients, the relative abundance of SCFAs-producing gut microbiota, their metabolic products SCFAs 138, 146, as well as the levels of lysine and nicotinic acid, exhibit statistically significant positive correlations with durable clinical benefits from ICIs therapy 147. These may potentially serve as biological markers for predicting the treatment response to ICIs.

### Preclinical evidence: Microbiota and their Derivatives effectively enhance antitumor immunotherapy responses

Beyond mere clinical correlation, interventional studies have further established the causal role of specific microbial taxa and their derivatives in modulating antitumor immune responses. Previous studies have demonstrated that oral supplementation with *A. muciniphila* or specific *Bifidobacterium bifidum* strains (e.g., *B. bif*_K57) effectively restores the efficacy of ICIs in tumor-bearing mouse models 132, 134. Compared to anti-PD-1 monotherapy, combination treatment with *A. muciniphila* promotes the recruitment of CCR9⁺CXCR3⁺CD4⁺ T cells into the TME, enhancing ICIs efficacy through an interleukin-12 (IL-12) -dependent mechanism 148. Conversely, *B. bif*_K57 significantly enhances antitumor lymphocyte infiltration (including CD4^+^/CD8^+^ T cells and activated NK cells) within the TME, elevates both CD8^+^ T/Treg and effector CD8^+^ T/Treg ratios, and upregulates key antitumor cytokines such as IFN-γ and IL-2 149, while suppressing TNF-α and IL-10, thereby mitigating chronic inflammation and restoring immune cell functionality 150,151. Notably, the antitumor efficacy of microbiota extends beyond viable bacteria, with their bioactive components playing equally critical roles. The extracellular vesicles derived from *Bifidobacterium bifidum* (*Bif.*BEVs) can enhance the efficacy of ICIs in NSCLC patients 152. Mechanistically, *Bif*.BEVs undergo dynamin-dependent endocytosis by tumor cells, activate the TLR4-NF-κB pathway, and upregulate PD-L1 expression, thereby promoting CD8⁺ T cell-dependent antitumor immunity. Additionally, similar to *B. bif*_K57, *Bif.*BEVs stimulate IFN-γ and IL-2 secretion. This functional conservation suggests that *Bifidobacterium bifidum* and its derivatives exert immunotherapeutic effects through convergent pathways. The probiotic *Clostridium butyricum* MIYAIRI 588, which is clinically used in NSCLC patients, enhances the efficacy of ICIs by promoting the colonization abundance of *Bifidobacterium* and suppresses tumor invasiveness by inhibiting Th17 cell differentiation, thereby exerting synergistic antitumor effects 145, 153. Microbial metabolites also serve as a pivotal immunomodulatory axis. Experimental evidence shows that SCFAs, including butyrate and propionate, can enhance the efficacy of antitumor immunotherapy by promoting the activity of intratumoral effector T cells 154, 155. Collectively, evidence from microbiota transplantation, administration of microbial structural components, and regulation of microbial metabolites establishes a causal role of gut microbiota and their derivatives in reshaping the TIME, highlighting their therapeutic potential as adjuvants to ICIs.

### Molecular mechanisms: Deep pathways of cross-organ immune regulation

**Figure 4:**
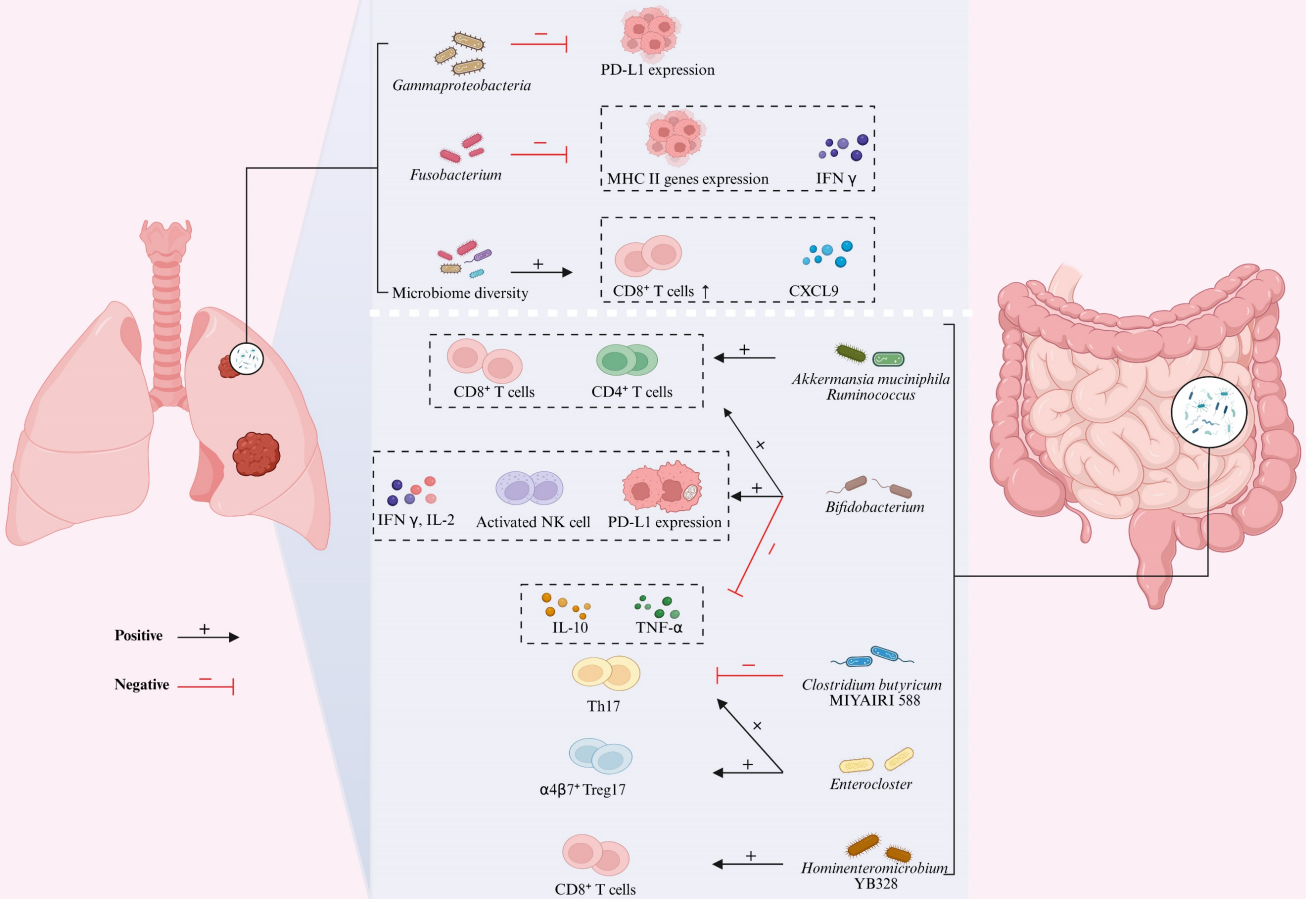
** Gut-Lung Axis Microbiota Modulates Immunotherapy Efficacy in Lung Cancer.** The gut-lung axis microbiota influences lung cancer progression through multi-layered immune regulatory mechanisms. Key microbial effects in the lung are characterized by: the negative correlation between *Gammaproteobacteria* abundance and PD-L1 expression, while *Fusobacterium* significantly suppresses IFN-γ and MHC class II gene expression; increased microbial α-diversity promotes CXCL9 secretion in the tumor microenvironment and CD8⁺ T cell recruitment, thereby enhancing PD-1 blockade efficacy. Key effects of gut microbiota are manifested as follows: *Akkermansia muciniphila* directly promotes the recruitment of tumor-infiltrating CD4⁺/CD8⁺ T cells; at low abundance, it synergizes with *Ruminococcaceae*, whose enrichment significantly expands circulating effector CD4⁺/CD8⁺ T cells and enhances tumor CD8⁺ T cell infiltration; the combination of *Bifidobacterium* with anti-PD-1 therapy simultaneously increases the numbers of tumor-infiltrating CD4⁺ T/CD8⁺ T/NK cells and the CD8⁺ T/Treg ratio, specifically upregulating IFN-γ/IL-2 while suppressing TNF-α/IL-10; *Clostridium butyricum* MIYAIRI 588 reduces tumor cell invasiveness by inhibiting Th17; the recolonization of *Enterocloster* in the gut drives the migration of α4β7⁺ Treg17 and IL-17-producing Th17 cells to tumor tissues, forming a complete microbiota-immune regulatory network. Figure 4 was created using BioRender and holds the appropriate license. C-X-C motif chemokine ligand 9, CXCL9; Interferon-γ, IFN-γ; Major histocompatibility complex, MHC; Mucosal addressin cell adhesion molecule 1, MAdCAM-1; Natural killer, NK; Interferon-gamma, IFN-γ; Programmed death-ligand 1, PD-L1 T-helper, Th17; Regulatory T cells, Tregs.

The molecular mechanisms underlying these therapeutic effects primarily involve multi-layered processes, including cross-organ migration of immune cells, activation of signaling pathways, and metabolic reprogramming (Figure 4). Research indicates that increased α-diversity of the lung microbiota shows a significant association with elevated C-X-C motif chemokine ligand 9 (CXCL9) secretion and enhanced CD8^+^ T cell infiltration in the tumor microenvironment (TME) 129. As a key chemokine, CXCL9 primarily mediates the directional migration of lymphocytes to tumor sites, thereby suppressing tumor growth 156. These findings suggest that the lung microbiota may regulate tumor sensitivity to ICIs by promoting CD8^+^ T cell recruitment via CXCL9-dependent mechanisms. Notably, a similar immunomodulatory mechanism exists in the gut microbiome, where patients with enrichment of *Ruminococcus* exhibit not only elevated frequencies of circulating effector CD4^+^ and CD8^+^ T cells but also significantly increased CD8^+^ T cell infiltration in tumor tissues 137. In-depth research has revealed that the gut microbiota acts as the “central regulator” of immune cell migration. Lin et al. 141 demonstrated that *Hominenteromicrobium* YB328 in the gut of NSCLC patients induces maturation of intestinal CD103^+^CD11b^-^ conventional dendritic cells (cDCs), enhancing their antigen-presenting capacity and costimulatory signaling, driving their migration to tumor-draining lymph nodes and subsequent infiltration into the TME. These cDCs locally activate tumor-specific CD8⁺ T cells while promoting PD-1 expression, thereby reshaping the local immune response and significantly improving PD-1 inhibitor efficacy 157. However, when the gut microbiota balance is disrupted, it can exert completely opposite immunomodulatory effects. For example, antibiotic-induced overgrowth of *Enterocloster* downregulates intestinal mucosal addressin cell adhesion molecule-1 (MAdCAM-1), leading to the aberrant migration of α4(CD49d) β7^+^ Th17 and Treg17 cells from the circulation to the gut and tumor tissues 144. Given the potent immunosuppressive function of these cells, their tumor infiltration significantly diminishes the therapeutic efficacy of ICIs 158.

**Figure 5:**
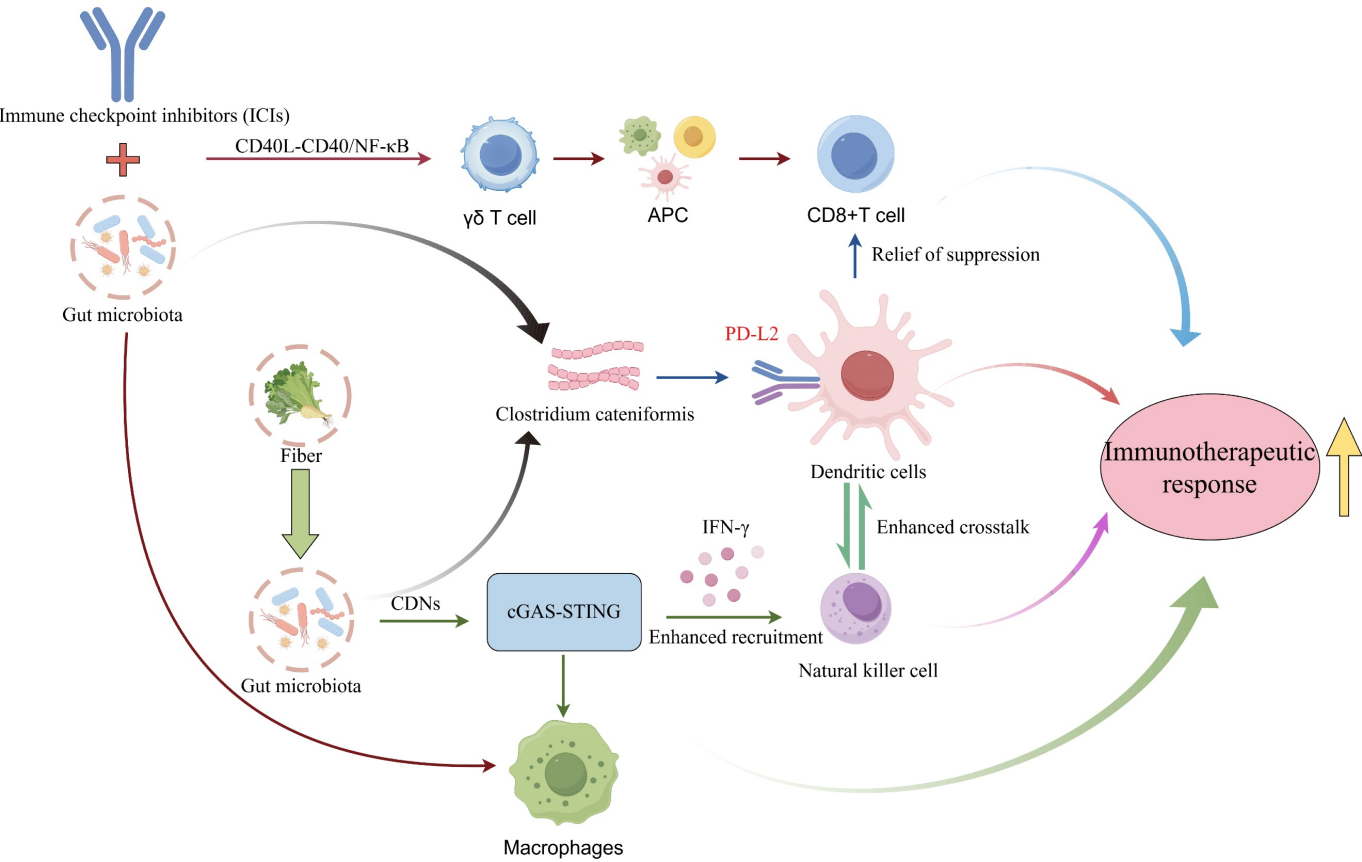
** Gut Microbiota Enhances Lung Cancer Immunotherapeutic Response via various Signaling Pathways.** Gut microbiota synergistically modulates the TIME via multiple core signaling cascades, thereby potentiating immunotherapeutic responses in lung cancer. Specifically, high-fiber diet-induced gut microbiota produces CDNs, which serve as natural agonists to activate the cGAS-STING pathway, promote IFNs secretion, recruit NK cells, reprogram macrophage polarization, enhance crosstalk between NK cells and DCs, and improve immune surveillance within the tumor microenvironment. Additionally, combined therapy with intact gut microbiota and ICIs activates the CD40L-CD40/NF-κB pathway, driving the phenotypic switch of macrophages from SPP1⁺ protumoral to CD74⁺ antigen-presenting states, facilitating the expansion of effector-memory CD8⁺ T cells while preventing their terminal exhaustion, and constructing a γδ T cell-APC-CD8⁺ T cell activation loop. Furthermore, beneficial gut commensals such as Clostridium cateniformis downregulate the expression of PD-L2 on DCs, blocking the immunoinhibitory interaction between PD-L2 and RGMb to relieve T cell suppression. These pathways form functional crosstalk via core immune cells including macrophages and DCs, ultimately synergistically enhancing lung cancer immunotherapeutic response and breaking tumor immune tolerance. Cyclic dinucleotides, CDNs; cyclic GMP-AMP synthase-stimulator of interferon genes, cGAS-STING; interferon, IFN; natural killer cells, NK cells; dendritic cells, DCs; immune checkpoint inhibitor, ICI; nuclear factor kappa-light-chain-enhancer of activated B cells, NF-κB; antigen-presenting cell, APC; programmed death ligand 2, PD-L2; repulsive guidance molecule B, RGMb; tumor immune microenvironment, TIME

Specific microbial species and their metabolites can directly activate antitumor immune responses by modulating key immunological signaling pathways (Figure 5). As a critical bridge connecting innate and adaptive immunity, activation of the cGAS-STING (cyclic GMP-AMP synthase-stimulator of interferon genes) pathway and subsequent robust production of type I interferons (IFN-I) is essential for breaking tumor immune tolerance 159. Research demonstrates that gut microbiota-derived cyclic dinucleotides (CDNs) under high-fiber dietary conditions serve as natural STING agonists, stimulating IFN-I production by tumor-infiltrating monocytes. This signaling axis not only recruits NK cells and reprograms macrophage polarization but also enhances crosstalk between NK cells and DCs, thereby significantly improving immune surveillance within the TME 160. Combined intact microbiota-ICI therapy enhances effector-memory CD8^+^ T cell expansion while preventing terminal exhaustion, and shifts macrophages from SPP1^+^ protumoral to CD74^+^ antigen-presenting states, establishing a γδ T cell-antigen-presenting cell (APC)-CD8^+^ T cell activation loop via CD40L-CD40/nuclear factor kappa-light-chain-enhancer of activated B cells (NF-κB) signaling 161. Although FMT is known to influence the efficacy of immunotherapy, treatment outcomes remain inconsistent. Emerging research reveals that beneficial gut commensals such as Clostridium cateniformis can downregulate PD-L2 expression on DCs. This alteration disrupts the immunoinhibitory PD-L2/repulsive guidance molecule B (RGMb) interaction, thereby relieving T-cell suppression and enhancing antitumor immune response 162.

The gut microbiota and its metabolic byproducts act as critical immunoregulatory agents that significantly shape the TME. Research by Zhu et al. 163 demonstrates that gut-derived *A. muciniphila* migrates via the bloodstream to colonize lung adenocarcinoma tissue, exerting antitumor effects. This activity likely stems from its crosstalk with intratumoral microbiota, which reprograms local metabolic pathways, particularly purine, glutathione, and arginine-proline metabolism 164. Microbial metabolites constitute deep immune regulatory mediators, and their produced small molecule signals can influence tumor immune responses through the gut-lung axis pathway 165, 166. It is noteworthy that SCFAs in lung tissue are primarily produced in large quantities by gut microbiota through fermentation of dietary fiber, while the contribution of lung microbes is relatively limited 167. Intestinal-derived SCFAs regulate the generation of Ly6C⁻ patrolling monocytes in the bone marrow through systemic circulation. These monocytes migrate to the lungs and differentiate into macrophages, which reduce neutrophil chemokine CXCL1 expression to blunt airway neutrophil recruitment during infection while enhancing CD8⁺ T cell responses, thereby limiting lung tissue pathology caused by inflammation 168. Emerging evidence demonstrates that butyrate enhances the antitumor immune response of cytotoxic CD8⁺ T cells through the IL-12 signaling pathway both *in vitro* and *in vivo* 169. Other SCFAs, such as sodium butyrate and sodium propionate, exhibit potential in suppressing lung cancer cell growth, triggering apoptosis, inducing cell cycle arrest, and modulating immune responses 154, 170. The gut-lung microbiota axis modulates antitumor immune responses through the aforementioned mechanisms. However, it must be emphasized that current mechanistic insights predominantly rely on murine models, lacking validation in human studies. Given the immunological, microbial, and pharmacological distinctions between murine and human systems, translational applications of current findings require further clinical validation.

## Clinical Translation Pathway of the Gut-Lung Microbiota Axis: From Prediction to Intervention

### Clinical Translational Pathways of the Gut-Lung Microbiota Axis

The clinical translational pathway of the gut-lung microbiota involves the construction of predictive models and the application of intervention strategies. Microbial marker studies demonstrate that a lung cancer prediction model based on the *Streptococcus* single microbial species showed an area under the curve (AUC) of 0.693-0.897, further research revealed that a combination prediction model constructed with three characteristic operational taxonomic units (OTU19/594/645) selected from this genus exhibited significantly improved efficacy, achieving an AUC of 0.701 27, 36. The two-bacteria synergistic level demonstrates that the diagnostic prediction model for lung cancer combining *Veillonella* and *Megasphaera* achieves an AUC of 0.880 55, while the more specific *Veillonella* and* Capnocytophaga* combination predicts squamous cell carcinoma (SCC) with an AUC of 0.860 171. Moreover, at the multi-dimensional integration level, breakthrough achievements have been presented. Zheng et al. 51 established an early diagnostic model by screening 13 gut OTUs using machine learning, achieving AUC values of 0.976 in the training set and 0.764 in the validation cohort. The Liu team developed a LUAD diagnosis model based on fungal features, ultimately incorporating 19 characteristic gut OTUs, with the predictive model achieving an AUC of 0.935 42. Extensive research that integrates microbial SCFAs, such as acetic acid and butyric acid 26, and tumor markers including neuron-specific enolase (NSE) 35, enhances the model's predictive performance to an AUC range of 0.959-0.993. Meanwhile, the incorporation of age and smoking factors attains an AUC of 0.882 56. As lung cancer prediction models undergo continuous optimization, these microbial biomarkers are being innovatively utilized for immunotherapy response prediction, thereby manifesting comprehensive clinical value spanning from diagnosis to treatment. Huang et al. 172 performed fecal microbiome sequencing before immunotherapy on 862 lung cancer patients and screened 26 characteristic fungi (primarily belonging to the *Ascomycota* phylum) through machine learning, establishing an immunotherapy response prediction model with AUC reaching 0.870. In responders, the abundance of 20 fungi such as *Trichophyton benhamiae* was significantly increased, whereas in non-responders, six fungi including *Pseudocercospora musae* were markedly enriched. The model predictions significantly correlated with patients' longer OS and PFS. Moreover, the model effectively distinguished the exhaustion level of CD8+ T cells and the expression of immune checkpoints, such as PD-1, CTLA-4, and T-cell immunoglobulin and mucin-domain containing-3 (TIM-3), within the TME. These molecules are associated with the response to immunotherapy 173, 174. Furthermore, integrating 20 fungal and 17 bacterial species, the study built a multi-kingdom microbial model with AUC of 0.890. Network analysis indicated that the core species Schizosaccharomyces octosporus modulates starch metabolic pathway, and its produced SCFAs may mediate anti-cancer effects 175-177. Yang et al. applied a support vector machine (SVM) machine learning model to predict the response efficacy to the combined chemotherapy and anti-PD-1 immunotherapy regimen in NSCLC patients by analyzing gut microbiome composition data. When adopting the top 20 most predictive microbial features at the genus level, the AUC was 0.763; upgrading to species-level features elevated the AUC to 0.855, indicating that higher classification accuracy can optimize predictive performance 178. Additionally, dysbiosis in the human body correlates with immune-related adverse events (irAEs). Lower gut microbial α diversity associates with increased risk of severe adverse events (≥ grade 4) 136^.^ Furthermore, abundances of specific microbes such as *Akkermansia*, *Lactobacillaceae*, and *Raoultella* associate with decreased risk of irAEs, while *Agathobacter* increases the risk of irAEs 21. In conclusion, this study demonstrates that disease diagnostic and prognostic models based on multi-species microbial signatures provide greater clinical value than those relying on single microbial strains. Microbiota-metabolomics integrated prediction models demonstrate considerable potential for lung cancer diagnosis and predicting immunotherapy efficacy. Nevertheless, their clinical validation remains inadequate, as current models are primarily developed using limited-scale cohorts from single-center or public databases with population homogeneity. This fundamentally restricts their external validity. Large-scale multicenter prospective validation studies are thus critically needed to establish their real-world clinical applicability.

### Clinical Intervention Potential of Microbiota

The clinical intervention potential of microbes has emerged as a key research focus in current studies. Accumulating evidence indicates that gut microbiome remodeling through FMT and probiotic supplementation can significantly enhance therapeutic response rates across various malignancies. In murine models, transplantation of fecal microbiota from either ICIs responders or non-responders among NSCLC patients into tumor-bearing mice revealed that responder-derived FMT induced substantial antitumor responses, whereas non-responder-derived FMT demonstrated limited therapeutic efficacy 20. Clinical investigations by Wu et al. 179 identified *Alistipes finegoldii* as a key microbial species associated with improved immunotherapy outcomes in multiple solid tumors, including NSCLC. Based on this discovery, a phase I/II clinical trial (NCT05557696) evaluating its therapeutic supplementation has been initiated, signifying a crucial transition toward clinical translation of microbiota-targeted interventions. Notably, the therapeutic efficacy of FMT in various cancers has been substantiated by multiple studies. Baruch et al. 180 demonstrated that melanoma patients receiving FMT from immunotherapy responders achieved a 30% ORR, with all treated subjects maintaining PFS for at least six months. Subsequent studies have further validated this approach in melanoma 181-183, colorectal cancer 184, and pan-cancer cohorts 185, confirming that fecal microbiota from healthy donors or treatment responders not only enhances immunotherapy efficacy and overcomes resistance but also significantly improves ORR and prolongs both PFS and OS, thereby establishing a novel paradigm for cancer immunotherapy. The safety profile of FMT remains a critical clinical concern, as evidenced by case reports of *Clostridium perfringens* infections post-FMT 186. Our systematic evaluation of adverse event (AE) data from the aforementioned studies reveals that 40% of investigations failed to report FMT-related AEs, while the remaining studies documented AE incidence rates ranging from 40% to 53.8% 182, 183, 185. Notably, the majority of AEs comprised mild gastrointestinal manifestations, including diarrhea, abdominal distension, and emesis, with no clustering of severe adverse events observed. Recent improvements in FMT safety primarily stem from the implementation of stringent donor screening protocols: the integration of sequencing and culture techniques to exclude samples containing pathogenic microorganisms has substantially mitigated infection risks. Compared to the complexity of FMT, oral probiotics (e.g., *Clostridium butyricum*, *Bifidobacterium*) demonstrate better clinical translatability owing to scalable production. Clinical evidence confirms their ability to potentiate immunotherapy, elevating response rates and PFS in lung, renal and urothelial carcinomas 133, 187-190. However, contradictory findings have emerged, as one retrospective melanoma study reported an unexpected association between probiotic use and impaired PFS. The discrepancy could stem from using non-specific commercial probiotics with limited immunomodulatory effects and an underpowered sample size 191. Notably, this study further indicates that baseline gut microbiota characteristics significantly influence probiotic efficacy, underscoring the need for microbial assessment prior to probiotic intervention in clinical practice. Furthermore, existing studies have investigated the efficacy-enhancing effects of clinically established postbiotic JK5G 192 and Microecosystem Therapy 4 (MET4) 193 in combination with immunotherapy: administration of JK5G has been shown to reduce the incidence of immune-related adverse events (irAEs), while MET4 significantly improves clinical benefit rates. Notably, no adverse effects associated with probiotic or postbiotic supplementation have been reported to date, demonstrating their favorable safety profile. In summary, modulating gut microbiota through FMT and specific probiotic supplementation can effectively enhance tumor immunotherapy response rates, overcome drug resistance, and prolong patient survival. Comparatively, probiotic formulations show greater clinical translational potential due to their favorable safety profile. However, significant limitations remain including inadequate FMT research in lung cancer, considerable variability in probiotic efficacy, insufficient sample sizes, and unclear strain specificity, along with unresolved safety concerns regarding FMT and incomplete mechanistic understanding of probiotic actions. Future research should prioritize screening high-efficacy bacterial strains with expanded validation efforts while establishing standardized microbiota assessment protocols to facilitate standardized clinical application of microbial-based therapies.

## Conclusion

As the leading cause of cancer incidence worldwide, lung cancer presents significant challenges in early prevention and prognosis improvement. The gut-lung microbiota axis has emerged as a pivotal hub in cross-organ communication, playing a critical role in disease progression and treatment response. Current evidence underscores that lung dysbiosis drives tumorigenesis through chronic inflammation and mucosal barrier disruption, whereas gut homeostasis and its metabolites can remodel the systemic immune microenvironment and regulate the bone marrow-lung axis, thereby enhancing antitumor surveillance and sensitivity to ICIs. However, the translation of these findings faces a critical bottleneck. The core regulatory mechanisms are predominantly derived from animal models, while direct human validation remains scarce. Although clinical studies have observed correlations between the abundance of specific microbiota (e.g., *Akkermansia muciniphila*) and therapeutic responses or irAEs, direct evidence remains lacking regarding how these functional microbes precisely modulate human immunity and how their metabolites directly activate CD8⁺ T cells *in vivo*. Consequently, future research must focus on "bridging the clinical evidence gap". Efforts should prioritize the integration of large-scale clinical cohorts, multi-omics approaches, and patient-derived organoid models to systematically decode the gut-lung interaction rules within the human context. Furthermore, developing high-sensitivity and high-specificity predictive models based on distinct microbiome signatures is essential. Ultimately, these advancements will pave the way for precise, microbiome-targeted intervention strategies, moving the field from correlative studies to clinical translation and offering innovative solutions to optimize ICIs regimens and improve patient outcomes in lung cancer.

## Supplementary Material

Supplementary figure and tables.

## Figures and Tables

**Table 1:** Respiratory microbiota diversity patterns in lung cancer patients stratified by clinical characteristics

Year	Sample number	Smoking (%)	Sample type	Analytical Method	Histology	TNM stage	Microbiota Community Diversity Discrepancy
2016 22	196	94.0	Lung tissues	16S rRNA	Lung cancer	I-II 67.3%, III-IV 32.7%	Significantly lower α-diversity in tumor vs. adjacent normal tissues.
2016 55	56	60.7	BALF	NGS	Lung cancer	I-II 33.3%, III-IV 66.7%	Significantly higher α-diversity in lung cancer group vs. benign mass-like lesion group.
2017 27	66	47.6	Bronchial brushing	16S rRNA	Lung cancer	I-II 25.0%, III-IV 66.7%	Significantly lower α-diversity in LC patients vs. healthy controls and distinct β-diversity in cancerous sites vs. paired contralateral non-cancerous sites.
2018 31	176	92.6	Lung tissues	16S rRNA	NSCLC	NA	Significantly higher α-diversity and distinct β-diversity in cancer vs. normal lung tissues.
2018 44	190	88.9	Bronchial brushing BALF	16S rRNA	Lung cancer	NA	β-Diversity differed significantly in BALF samples between healthy individuals and LC patients.
2018 32	40	100.0	Lung tissues	16S rRNA	Lung cancer	I-II 52.5%, III-IV 10.0%	Significantly higher α-diversity and distinct β-diversity in LC patients vs. emphysema-only patients.
2019 56	150	56.0	BALF	MGS	Lung cancer	I-II 45.7%, III-IV 53.1%	Significantly lower LRT richness in LC patients vs. healthy controls.
2019 39	92	57.6	BWF, sputum	16S rRNA	NSCLC	NA	No significant differences
2020 45	90	88.9	Saliva, BALF lung tissues	16S rRNA	NSCLC	I-II 55.6%, III-IV 44.4%	NSCLC salivary microbiota had distinct β-diversity vs. lung specimens (BALF/tumor/adjacent/distal).
2020 36	125	78.1	Saliva, BALF	16S rRNA ITS	Lung cancer	NA	Significantly higher α-diversity in saliva and BALF microbiota of LC patients than non-cancer controls. Affected bronchi showed distinct β-diversity of fungi compared to the contralateral bronchi.
2021 25	47	72.3	BALF	MG-Seq	NSCLC	I-II 71.9%, III-IV 28.1%	Significantly lower α-diversity in BALF samples from NSCLC patients vs. non-cancer controls.
2021 38	83	90.4	Bronchial brushing	16S rRNA	Lung cancer	I-II 26.0%, III-IV 73.0%	β-Diversity significantly differed by tumor type (SCLC vs. NSCLC), TNM stage (I-IIIA vs. IIIB-IV stage), and survival duration (short-term [<1 year] vs. long-term [>1 year] ).
2022 37	8	0.0	Bronchial brushingBALF	16S DNA	Lung cancer	NA	β-Diversity differed significantly between tumor regions and non-tumor regions.
2022 34	78	NA	BALF	NGS	Lung cancer	NA	Significantly higher α-diversity in LC patients vs. tuberculosis and pneumonia patients.
2022 35	60	53.3	BALF	MGS	Lung cancer	I-II 17.2%, III-IV 62.1%	Significantly higher α-diversity in BALF samples from LC patients vs. benign disease controls.
2022 29	85	47.1	Sputum	16S rRNA	NSCLC	I-II 26.0%, III-IV 60.0%	Significantly lower α-diversity and distinct β-diversity in advanced-stage (III-IV) vs. early-stage (I-II) NSCLC patients.
2022 23	162	60.5	Lung tissues	16S rRNA	NSCLC	I-II 82.5%, III-IV 17.5%	Significantly lower α-diversity and distinct β-diversity in NSCLC tissues vs. normal tissues.
2022 30	1306	90.4	Oral swab	16S rRNA	Lung cancer	NA	Lower α-diversity was associated with higher LC risk.
2023 46	38	15.8	BALF	MG-Seq	NSCLC	I-II 86.4%, III-IV 13.6%	Significantly higher α-diversity and distinct β-diversity in NSCLC patients vs. non-NSCLC patients.
2023 26	56	55.6	BALF	MGS	Lung cancer	I-II 7.4%, III-IV 66.7%	Significantly lower α-diversity and distinct β-diversity in tumor-burden segments vs. ipsilateral non-tumor sites.
2023 40	71	78.3	Saliva, BALF	16S rRNA	Lung cancer	NA	No significant differences
2023 24	52	19.0	Oral swab, BALF Lung tissues	ITS, MG-Seq	NSCLC	I-II 88.0%, III-IV 12.0%	Significantly lower α-diversity in tumor tissues vs. non-tumor tissues.
2024 28	116	53.5	Lung tissues	16S rRNA	Lung cancer	NA	Significantly lower α-diversity and distinct β-diversity in recurrent vs. non-recurrent LC patients.
2024 33	369	NA	Lung tissues	16S rRNA	NSCLC	NA	Significantly higher bacterial α-diversity and distinct β-diversity in NSCLC tumor tissue vs. lung parenchyma tissues.

* BALF, bronchoalveolar lavage fluid; BWF, bronchial washing fluid; ITS, internal transcribed spacer; LC, lung cancer; LRT, lower respiratory tract; MGS, shotgun metagenomics sequencing; MG-Seq, metagenomic sequence; NGS, next-generation sequencing; NSCLC, non-small cell lung cancer; sMPLC, synchronous multiple primary lung cancer; SCLC, small cell lung cancer; TNM, tumor node metastasis; WGS, whole genome sequencing.

**Table 2:** Gut microbiota diversity patterns in lung cancer patients stratified by clinical characteristics

Year	Sample number	Smoking (%)	Sample type	Analytical Method	Histology	TNM stage	Microbiota Community Diversity Discrepancy
2018 48	82	53.7	Stool	16S rRNA	Lung cancer	I-II 56.1%, III-IV 43.9%	β-diversity significantly differed in LC patients vs. healthy controls.
2019 49	60	NA	Stool	16S rRNA	Lung cancer	I-II 16.6%, III-IV 83.4%	β-diversity significantly differed in LC patients vs. healthy controls.
2019 41	46	NA	Stool	16S rRNA	Lung cancer	NA	Significantly lower α-diversity and distinct β-diversity in LC patients vs. healthy controls.
2020 51	107	2.4	Stool	16S rRNA	Lung cancer	I-II 100.0%	β-diversity significantly differed in LC patients vs. healthy controls.
2021 52	81	NA	Stool	16S rRNA	Lung cancer	I-II 30.8%, III-IV 67.9%	β-diversity significantly differed in LC patients vs. healthy controls.
2022 57	58	15.5	Stool	16S rRNA	NSCLC	I-II 95.7%, III-IV 4.3%	No significant difference.
2023 42	299	NA	Stool	ITS	LUAD	I-II 97.2%, III-IV 2.8%	Increased α- and β-diversity of gut fungal microbiome in LUAD vs. healthy controls.
2023 53	78	30.8	Stool	16S rRNA	NSCLC	I-II 100.0%	β-diversity significantly differed in early-stage NSCLC patients and healthy controls.
2024 43	83	18.0	Stool	16S rRNA	Lung cancer	I-II 90.9%, III-IV 9.1%	Significantly higher α-diversity and distinct β-diversity in LC with moderate/severe (vs. mild/normal) ventilation dysfunction.β-diversity significantly differed in LC patients vs. benign pulmonary disease patients.

* ITS, internal transcribed spacer; LC, lung cancer; LUAD, lung adenocarcinoma; NSCLC, non-small cell lung cancer; TNM, tumor node metastasis.

**Table 3:** Microbiota modulates response to immunotherapy in lung cancer patients.

Year	Microbial origin	ICIs therapy	Microbes beneficial for antitumor therapy	Clinical benefit
2021 130	Lung	PD-1/PD-L1 inhibitors	*Veillonella dispar*	Increased rates of CR, PR and SD.
2021 131	Lung	PD-1/PD-L1 inhibitors	*Gammaproteobacteria*	Decreased PFS and OS.
2022 129	Lung	PD-1/PD-L1 inhibitors	*Bacteroidetes*	Increased rates of PR and SD.
2024 99	Lung	ICB-monotherapy	*Fusobacterium*	Decreased PFS and OS.
2017 132	Gut	PD-1/PD-L1 inhibitors	*Akkermansia muciniphila*	Increased rates of PR, SD, and prolonged PFS.
2020 133	Gut	PD-1/PD-L1 inhibitors	*Clostridium butyricum* MIYAIRI 588	Prolonged PFS and OS.
2020 137	Gut	PD-1/PD-L1 inhibitors	*Ruminococcaceae* UCG 13 and *Agathobacter*	Prolonged PFS and OS, improved ORR.
2021 134	Gut	Chemo-immunotherapy combinations	*Bifidobacterium bifidum*	Increased rates of PR.
2022 135	Gut	PD-1/PD-L1 inhibitors	*Akkermansia muciniphila*	Increased rates of CR/PR, and prolonged OS
2024 138	Gut	PD-1/PD-L1 inhibitors	*Faecalibacterium*	Increased rates of CR and PR
2025 136	Gut	PD-1/PD-L1 inhibitors, CTLA-4 inhibitors, and chemotherapy	*Fusicatenibacter*, *Butyricicoccus* and *Blautia*	Prolonged OS
2025 152	Gut	PD-1/PD-L1 inhibitors	*Bif*.BEVs	Tumor burden reduction
2025 141	Gut	PD-1/PD-L1 inhibitors	*Hominenteromicrobium* YB328	Prolonged PFS
2025 178	Gut	Chemo-immunotherapy combinations	*Faecalibacterium* and* Subdoligranulum*	Prolonged PFS

* CTLA-4, cytotoxic T-lymphocyte-associated protein 4; CR, complete response; ICB, immune checkpoint blockade; ORR, objective response rates; OS, overall survival; PD-1, programmed death 1; PD-L1, programmed death-ligand 1; PR, partial response; PFS, progression-free survival; SD, stable disease.
