# Supplementary material for: Gut-Lung Microbiota Axis Shapes the Immune Microenvironment and Immunotherapeutic Response in Lung Cancer
Source: Int J Biol Sci. 2026 Feb 1;22(5):2265–84. doi: 10.7150/ijbs.126977 (PMC12965067; doi:10.7150/ijbs.126977)
Supplement: Supplementary file 1 — Supplementary figure and tables. [file ijbsv22p2265s1.pdf]

Supplementary table 1. Summary table of respiratory microbiota diversity patterns in lung cancer patients.

Year	Sample number	Smoking (%)	Sample type	Analytical Method	Histology	TNM stage	α-diversity	β-diversity
2016 [22]	196	94.0	Lung tissues	16S rRNA	Lung cancer	I-II 67.3%, III-IV 32.7%	↓	NA
2016 [55]	56	60.7	BALF	NGS	Lung cancer	I-II 33.3%, III-IV 66.7%	↑	No significant differences
2017 [27]	66	47.6	Bronchial brushing	16S rRNA	Lung cancer	I-II 25.0%, III-IV 66.7%	↓	Significant differences
2018 [31]	176	92.6	Lung tissues	16S rRNA	NSCLC	NA	↑	Significant differences
2018 [44]	190	88.9	Bronchial brushing BALF	16S rRNA	Lung cancer	NA	NA	Significant differences
2018 [32]	40	100.0	Lung tissues	16S rRNA	Lung cancer	I-II 52.5%, III-IV 10.0%	↑	Significant differences
2019 [56]	150	56.0	BALF	MGS	Lung cancer	I-II 45.7%, III-IV 53.1%	↓	No significant differences
2019 [39]	92	57.6	BWF, sputum	16S rRNA	NSCLC	NA	No significant differences	No significant differences
2020 [45]	90	88.9	Saliva, BALF lung tissues	16S rRNA	NSCLC	I-II 55.6%, III-IV 44.4%	NA	Significant differences
2020 [36]	125	78.1	Saliva, BALF	16S rRNA ITS	Lung cancer	NA	↑	Significant differences
2021 [25]	47	72.3	BALF	MG-Seq	NSCLC	I-II 71.9%, III-IV 28.1%	↓	No significant differences
2021 [38]	83	90.4	Bronchial brushing	16S rRNA	Lung cancer	I-II 26.0%, III-IV 73.0%	No significant differences	Significant differences
2022 [37]	8	0.0	Bronchial brushing BALF	16S DNA	Lung cancer	NA	No significant differences	Significant differences
2022 [34]	78	NA	BALF	NGS	Lung cancer	NA	↑	No significant differences
2022 [35]	60	53.3	BALF	MGS	Lung cancer	I-II 17.2%, III-IV 62.1%	↑	No significant differences
2022 [29]	85	47.1	Sputum	16S rRNA	NSCLC	I-II 26.0%, III-IV 60.0%	↓	Significant differences
2022 [23]	162	60.5	Lung tissues	16S rRNA	NSCLC	I-II 82.5%, III-IV 17.5%	↓	Significant differences
2022 [30]	1306	90.4	Oral swab	16S rRNA	Lung cancer	NA	↓	No significant differences
2023 [46]	38	15.8	BALF	MG-Seq	NSCLC	I-II 86.4%, III-IV 13.6%	↑	Significant differences
2023 [26]	56	55.6	BALF	MGS	Lung cancer	I-II 7.4%, III-IV 66.7%	↓	Significant differences
2023 [40]	71	78.3	Saliva, BALF	16S rRNA	Lung cancer	NA	No significant differences	No significant differences

2023 [24]	52	19.0	Oral swab, BALF Lung tissues	ITS, MG-Seq	NSCLC	I-II 88.0%, III-IV 12.0%	↓	NA
2024 [28]	116	53.5	Lung tissues	16S rRNA	Lung cancer	NA	↓	Significant differences
2024 [33]	369	NA	Lung tissues	16S rRNA	NSCLC	NA	↑	Significant differences

* This table summarizes key aspects including patient baseline characteristics, sample types, cancer subtypes, sequencing methods, and reported α-/β-diversity trends, to facilitate clear comparisons across different studies. BALF, bronchoalveolar lavage fluid; BWF, bronchial washing fluid; ITS, internal transcribed spacer; LC, lung cancer; LRT, lower respiratory tract; MGS, shotgun metagenomics sequencing; MG-Seq, metagenomic sequence; NGS, next-generation sequencing; NSCLC, non-small cell lung cancer; sMPLC, synchronous multiple primary lung cancer; SCLC , small cell lung cancer; TNM, tumor node metastasis; WGS, whole genome sequencing.

Supplementary table 2. Summary table of gut microbiota diversity patterns in lung cancer patients.

**Year**	**Sample number**	**Smoking (%)**	**Sample type**	**Analytical Method**	**Histology**	**TNM stage**	**α-diversity**	**β-diversity**
2018 [48]	82	53.7	Stool	16S rRNA	Lung cancer	I-II 56.1%, III-IV 43.9%	NA	Significant differences
2019 [49]	60	NA	Stool	16S rRNA	Lung cancer	I-II 16.6%, III-IV 83.4%	No significant differences	Significant differences
2019 [41]	46	NA	Stool	16S rRNA	Lung cancer	NA	↓	Significant differences
2020 [51]	107	2.4	Stool	16S rRNA	Lung cancer	I-II 100.0%	No significant differences	Significant differences

2021 [52]	81	NA	Stool	16S rRNA	Lung cancer	I-II 30.8%, III-IV 67.9%	No significant differences	Significant differences
2022 [57]	58	15.5	Stool	16S rRNA	NSCLC	I-II 95.7%, III-IV 4.3%	No significant differences	No significant differences
2023 [42]	299	NA	Stool	ITS	LUAD	I-II 97.2%, III-IV 2.8%	↑	Significant differences
2023 [53]	78	30.8	Stool	16S rRNA	NSCLC	I-II 100.0%	No significant differences	Significant differences
2024 [43]	83	18.0	Stool	16S rRNA	Lung cancer	I-II 90.9%, III-IV 9.1%	No significant differences	Significant differences

* This table summarizes key aspects including patient baseline characteristics, sample types, cancer subtypes, sequencing methods, and reported α-/β-diversity trends, to facilitate clear comparisons across different studies. ITS, internal transcribed spacer; LC, lung cancer; LUAD, lung adenocarcinoma; NSCLC, non-small cell lung cancer; TNM, tumor node metastasis.

Supplementary table 3. Differential respiratory and lung microbial composition between lung cancer patients and healthy controls.

**Year**	**Sample number**	**Sample type**	**Differential microbial taxa**
2016 [22]	196	Lung tissues	*Thermus* was enriched in stage IIIB-IV patients, whereas *Legionella* predominated in metastatic cases.
2016 [55]	56	BALF	*Firmicutes*, *Veillonella*, and *Megasphaera* were enriched in LC patients
2017 [27]	66	Bronchial brushing	*Streptococcus* was significantly enriched in LC patients, while *Staphylococcus* predominated in healthy controls.
2018 [31]	176	Lung tissues	*Acidovorax*, *Klebsiella*, *Rhodoferax*, and *Anaerococcus* were enriched in LUSC patients.
2018 [44]	190	Bronchial brushing, BALF	*Streptococcus*, *Prevotella* and *Veillonella* were enriched in LC patients
2018 [32]	40	Lung tissues	*Aggregatibacter*, *Escherichia/Shigella*, *Haemophilus* and *Neisseria* were enriched in LC patients, while *Acinetobacter* and *Acidovorax* predominated in healthy controls.
2019 [56]	150	BALF	*Prevotella*, *Propionibacterium*, *Streptococcus*, *Rothia*, *Haemophilus* and *Pseudomonas* were enriched in LC patients.

2019 [39]	92	BWF, sputum	*Streptococcus* decreased in metastatic LUAD, whereas *Veillonella* and *Rothia* increased in metastatic LUSC.
2020 [45]	90	Saliva, BALF, lung tissues	*Firmicutes* dominated in NSCLC BALF/saliva, while *Proteobacteria* predominated in tumor tissues.
2020 [36]	125	Saliva, BALF	*Streptococcus*, *Rothia*, *Gemella* and *Lactobacillus* were enriched in the saliva of LC patents, while *Streptococcus*, *Prevotella*, *Blautia*, *Veillonella* and *Malassezi*a dominated in BALF of LC patients.
2021 [25]	47	BALF	*Lactobacillus rossiae*, *Burkholderia mallei* and *Bacteroides pyogenes*, et al. were enriched in NSCLC patients.
2021 [38]	83	Bronchial brushing	*Veillonella*, *Prevotella*, and *Streptococcus* were significantly enriched in stage III-IV LC patients.
2022 [59]	128	Lung tissues	*Aspergillus* and *Agaricomycetes* were significantly enriched in tumor tissues obtained from smoking-associated LC patients.
2022 [60]	100	Lung tissues	*Blastomyces* was enriched in LUSC patients.
2022 [37]	8	Bronchial brushing, BALF	*Actinobacteria*, *Clostridium*, *Fusobacterium*, and *Rothia* were enriched in the airways of tumor-affected lung lobes in patients with sMPLC. Among these, *Fusobacterium*, *Leptotrichia*, and *Rothia* exhibited preferential colonization in the mucosal layer of tumor-associated airways.
2022 [34]	78	BALF	*Neisseria*, *Megamonas*, *Fusobacterium*, *Phenylobacterium* and *Lautropia* were enriched in LC patients.
2022 [35]	60	BALF	*Achromobacter*, *Chryseobacterium*, *Herbaspirillum*, *Pedobacter*, *Thermomonas*, *Undibacterium*, *Caulobacter*, *Novosphingobium*, *and Dechloromonas* were enriched in LC patients, while *Prevotella* predominated in healthy controls.
2022 [29]	85	Sputum samples	*Granulicatella* and *Actinobacillus* were enriched in early-stage (I-II) patients, while *Actinomyces* dominated advanced-stages (III-IV). *Parvimonas* was significantly enriched in LUAD patients with EGFR mutation.
2022 [23]	162	Lung tissues	*Romboutsia*, *Christensenellaceae R-7 group*, *Novosphingobium*, *Acinetobacter*, *Rhizobium*, and *Prevotella* were enriched in NSCLC tumor tissues; *Staphylococcus*, *Burkholderiaceae family*, and *Cutibacterium* predominantly colonized normal lung tissues; *Staphylococcus*, the *Burkholderia-Caballeronia-Paraburkholderia*, and *Peptoniphilus* were enriched in advanced-stage (III-IV) NSCLC patients; *Haemophilus influenzae* was overrepresented in LUSC subgroups; *Stenotrophomonas*, *Bacteroides*, and *Peptoniphilus* were enriched in NSCLC patients with recurrence.
2022 [30]	1306	Oral swab	*Abiotrophia*, *Lactobacillus*, *Streptococcus*, and *Peptoniphilus* enrichment positively correlated with lung cancer risk.
2023 [46]	38	BALF	*Alternaria arborescens* was significantly enriched in NSCLC patients.
2023 [26]	56	BALF	*Campylobacter*, *Enterobacter*, *Debaryomyces*, and *Fusobacterium* were enriched in lung segments with malignant burden, while *Bacillus*, *Klebsiella*, and *Acinetobacter* predominated in normal lung segments.
2023 [40]	71	Saliva, BALF	*Carnobacterium* and *Brucella* were significantly enriched in the BALF of cancerous sites, whereas *Prevotella*, *Prevotella 7*, and *Prevotella oralis* predominated in the healthy sites. *Gemella sanguinis* and *Streptococcus intermedius* were exclusively isolated from the BALF of NSCLC patients. *Bacillus* and *Castellaniella* were enriched in the BALF of LUAD, while *Brucella* was associated with LUSC.
2023 [24]	52	Oral swab, BALF, lung tissues	*Aspergillus sydowii* was the enriched fungus in NSCLC tissues.

2024 [28]	116	Lung tissues	*Roseburia* and *Blautia* were significantly enriched in LC patients with early recurrence and promote metastasis.
2024 [33]	369	Lung tissues, stool	*Prevotella 7*, *Leptotrichia*, *Alloprevotella*, and *Cutibacterium* were enriched in NSCLC cancer tissues.

* BALF, bronchoalveolar lavage fluid; BWF, bronchial washing fluid; EGFR, epidermal growth factor receptor; LC, lung cancer; LUSC, lung squamous carcinoma; LUAD, lung adenocarcinoma; NSCLC, non-small cell lung cancer; sMPLC, synchronous multiple primary lung cancer.

Supplementary table 4. Gut microbial dysbiosis in lung cancer patients compared to healthy controls.

Year	Sample number	Sample type	Differential microbial taxa
2018 [48]	82	Stool	*Bacteroides*, *Veillonella*, and *Fusobacterium* were enriched in LC patients,while *Escherichia*, *Kluyvera*, *Faecalibacterium*, *Enterobacter*, and *Dialister* predominated in healthy controls.
2019 [49]	60	Stool	*Enterococcus* were enriched in LC patients, while *Actinobacteria* and *Bifidobacterium* predominated in healthy controls.
2019 [41]	46	Stool	*Megasphaera* and *Phascolarctobacterium* were enriched in LC patients, while *Coprococcus* predominated in healthy controls.
2020 [51]	107	Stool	*Bacteroidetes* and *Proteobacteria* were enriched in LC patients, whereas *Firmicutes* and *Actinobacteria* showed depletion.
2021 [52]	81	Stool	*Actinomyces*, *Veillonella*, *Megasphaera*, *Enterococcus* and *Clostridioides* were enriched in LC patients.
2022 [57]	58	Stool	*Prevotella*, *Roseburia*, and *Gemmiger* were enriched in NSCLC patients.
2023 [42]	299	Stool	*Basidiomycota*, *Mortierellomycota*, *Chytridiomycota*, *Saccharomyces*, *Aspergillus*, *Apiotrichum* and *Penicillium* were enriched in LUAD patients, while *Ascomycota*, *Vanrija*, *Pichia*, and *Trichosporon* were markedly lower in the LUAD group.
2023 [53]	78	Stool	*Agathobacter*, *Blautia*, *Clostridium*, and *Muribaculacea* were enriched in the early-stage (I-II) NSCLC patients.
2023 [61]	282	Rectal swab	*Streptococcus anginosus* enriched in the rectal swab of LUAD, and *Corynebacterium aurimucosum* dominated in LUSC.
2024 [43]	83	Stool	*Bacteroides* and *Veillonella* were enriched in LC patients, while *Blautia* and *Bifidobacterium* predominated in healthy controls.
2024 [33]	369	Stool	*Bacteroidota*, *Desulfobacterota*, *Bacteroides*, *Alistipes* and *CAG-873* were enriched in the stool of NSCLC patients, whereas *Blautia*, *Romboutsia*, *Clostridium sensu stricto 1* and *Fusicatenibacter* dominated in healthy controls.

* BALF, bronchoalveolar lavage fluid; BWF, bronchial washing fluid; EGFR, epidermal growth factor receptor; LC, lung cancer; LUSC, lung squamous carcinoma; LUAD, lung adenocarcinoma; NSCLC, non-small cell lung cancer; sMPLC, synchronous multiple primary lung cancer

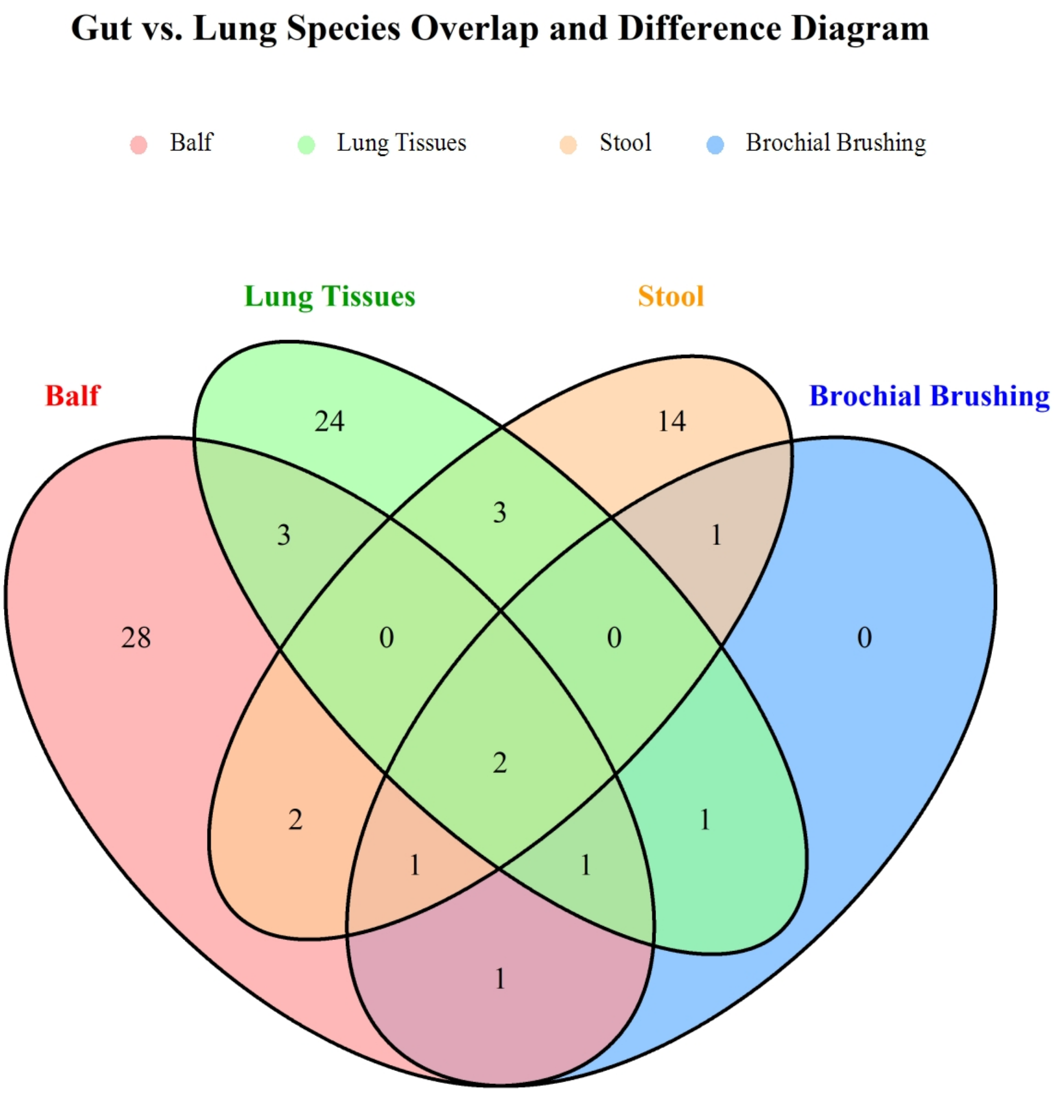

Supplementary figure 1: Venn analysis of overlapping and unique lung cancer-associated microbiota (at genus level) across lung and gut ecosystems. Microbial distribution analysis revealed that samples from different anatomical sites were enriched with specific lung cancer-associated microbes, while g_*Prevotella* and g_*Veillonella* were concurrently detected in both lung and gut samples.
